# Type 2 Diabetes Mellitus Increases the Risk of Late-Onset Alzheimer’s Disease: Ultrastructural Remodeling of the Neurovascular Unit and Diabetic Gliopathy

**DOI:** 10.3390/brainsci9100262

**Published:** 2019-09-29

**Authors:** Melvin R. Hayden

**Affiliations:** 1 Diabetes and Cardiovascular Center, University of Missouri School of Medicine, Columbia, MO 65212, USA; mrh29pete@gmail.com; Tel.: +1-573-346-3019; 2 Division of Endocrinology and Metabolism, Department of Medicine, University of Missouri, Columbia, MO 65212, USA

**Keywords:** aging, Alzheimer’s disease, brain insulin resistance, *db/db* diabetic mouse model, diabetic cognopathy, insulin resistance, metabolic syndrome, mixed dementia, obesity, type 2 diabetes mellitus

## Abstract

Type 2 diabetes mellitus (T2DM) and late-onset Alzheimer’s disease–dementia (LOAD) are increasing in global prevalence and current predictions indicate they will only increase over the coming decades. These increases may be a result of the concurrent increases of obesity and aging. T2DM is associated with cognitive impairments and metabolic factors, which increase the cellular vulnerability to develop an increased risk of age-related LOAD. This review addresses possible mechanisms due to obesity, aging, multiple intersections between T2DM and LOAD and mechanisms for the continuum of progression. Multiple ultrastructural images in female diabetic *db/db* models are utilized to demonstrate marked cellular remodeling changes of mural and glia cells and provide for the discussion of functional changes in T2DM. Throughout this review multiple endeavors to demonstrate how T2DM increases the vulnerability of the brain’s neurovascular unit (NVU), neuroglia and neurons are presented. Five major intersecting links are considered: i. Aging (chronic age-related diseases); ii. metabolic (hyperglycemia advanced glycation end products and its receptor (AGE/RAGE) interactions and hyperinsulinemia-insulin resistance (a linking linchpin); iii. oxidative stress (reactive oxygen–nitrogen species); iv. inflammation (peripheral macrophage and central brain microglia); v. vascular (macrovascular accelerated atherosclerosis—vascular stiffening and microvascular NVU/neuroglial remodeling) with resulting impaired cerebral blood flow.

## 1. Introduction

The human central nervous system culminates in the brain that contains a virtual galaxy of cells within a universe. Estimates of brain cell numbers includes: 100 billion neurons [1], 85–100 billion neuroglia (astrocytes, microglia and oligodendrocytes) [2] and 0.15 quadrillion synapses [3], which makes our brain cell numbers somewhat comparable to that of our own Milky Way galaxy. Penetrating arteries, arterioles and capillary neurovascular unit(s) (NVU) course throughout the brain parenchyma, which consist of endothelial cell(s) (EC), vascular smooth muscles cells, capillary pericyte(s) (Pcs) and neuroglia. It has been estimated that there are approximately 100 million capillary NVUs that course through the brain for approximately 400 miles, which allows for a vast surface area of approximately 20 square meters to provide cellular, molecular and ion transport to and from the brain to the systemic circulation [4]. Importantly, each of these cells have multiple unique ultrastructural identifying forms and functions [5,6,7,8,9,10]. Thus, the brain parenchyma and its vascular supply are the structural and functional substrate of the mind, which allow each of us to have a unique brain print. 

Type 2 diabetes mellitus (T2DM) and late-onset Alzheimer’s disease–dementia (LOAD) are increasing in global prevalence and current predictions indicate they will increase over the coming decades as our global society continues to age. Since T2DM may increase the risk of LOAD, it is important to understand the relationship between these two disparate diseases. Age-related T2DM shares multiple common metabolic, hormonal, inflammatory, vascular, genetic, environmental and structural remodeling stress–risk factors, which may allow for increased cellular vulnerability to develop age-related LOAD. Importantly, the arteriole and capillary neurovascular unit(s) (NVU) provide the neurovascular coupling necessary for functional hyperemia in order to provide the necessary cerebral blood flow of regionally active neurons. 

This review discusses the importance of societal aging, insulin resistance, hyperinsulinemia, hypothalamic pituitary axis (HPA) dysfunction, risk factors between T2DM and LOAD and the continuum of progression from obesity to insulin resistance, metabolic syndrome (MetS), T2DM and importantly to LOAD. Further, these discussions lead to the central focus of this review, which is the ultrastructural aberrant remodeling changes of the capillary NVU including both the mural and glia cells.

### 1.1. T2DM, Late-Onset Alzheimer’s Disease–Dementia (LOAD) and Societal Aging 

T2DM is a heterogeneous, multifactorial, polygenic, endocrine, metabolic, chronic and age-related disease characterized by obesity, insulin resistance (IR) and hyperglycemia. Further, T2DM is a result of the relative or complete impairment of insulin actions and signaling, and is associated with a progressive decline in pancreatic beta cell function [5,6,7,8,9,10,11,12,13,14,15,16]. 

**Figure 1:**
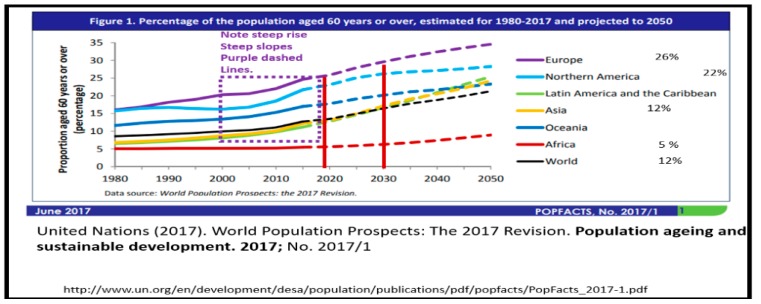
Graphical representation of the percent of the global population age 60 or greater. This figure is divided into global regions and age groups from the United Nations (2017) population aging and sustainable development—the United Nations [17].

We live in a time where there exists one of the oldest-living global populations [16] and there is evidence that suggests the number of older people in our global population is going to increase in the coming decades (Figure 1) [17]. 

This aging population is largely due to the aging baby boom generation that benefited from the advancement of modern medicine including immunizations, antibiotics and cerebrovascular–cardiovascular care including hypertension and cholesterol control, coronary artery stenting, bypass grafting and stroke care. Additionally, this global aging generation resulted from the high birth rates following World War II and is partially responsible for driving this global increase. As this aging generation continues to age, they also undergo an increase in age-related loss of skeletal muscle mass with increasing skeletal muscle insulin resistance. Moreover, there is an associated decrease in physical activity and increased consumption of compact calorie dense Western diets, which contribute to the parallel global increase of obesity, T2DM and LOAD. Globally, there are 425 million patients estimated to have T2DM, and by 2040 it is expected that this figure will rise to 642 million [18,19,20]. Importantly, it has been recently estimated that as this generation ages, 13.5 million individuals in the United States may manifest LOAD by the year 2050 [21] and the current lifetime risk of a 65-year-old individual for LOAD is now estimated at 10.5% [22]. In fact, as T2DM and LOAD merge in this aging society they may form a “bottleneck” of senior citizens with co-occurrence dementias. This conundrum will undoubtedly create a strain on our healthcare system, a financial burden to our society and much stress to individual caregivers and families.

### 1.2. Aging, Obesity, Insulin Resistance, Metabolic Syndrome (MetS)

**Figure 2:**
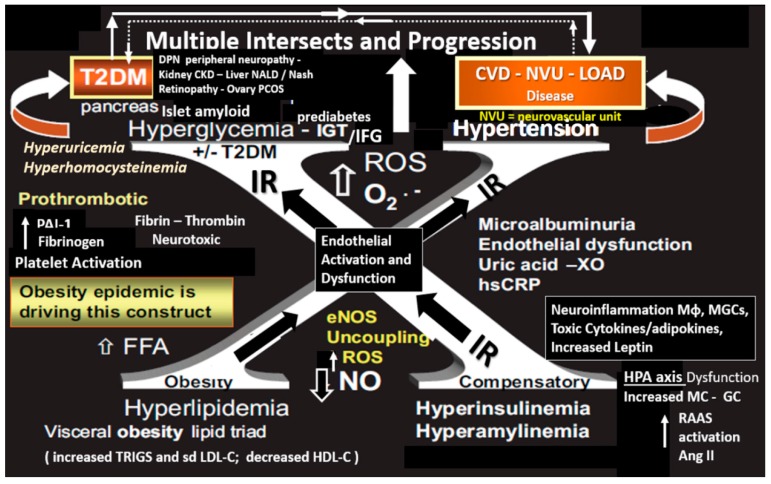
Metabolic syndrome with multiple intersects and progression. This image illustrates the metabolic syndrome utilizing the letter “X” in order to honor Reaven’s original term Syndrome X, who championed the concept that resistance to insulin-mediated glucose disposal was a characteristic of patients with type 2 diabetes mellitus (T2DM), which was later termed the metabolic syndrome (MetS). Note the four different hyper-conditions associated with the MetS on each arm of the large X in addition to hyperuricemia and hyperhomocysteinemia, which may synergistically act with hyperglycemia to generate increased oxidative/nitrosative stress. This figure depicts peripheral insulin resistance (IR); however, there may be a contribution to central brain insulin resistance (BIR). CKD = chronic kidney disease; CVD = cardio-cerebrovascular disease; DPN = diabetic peripheral neuropathy; eNOS = endothelial nitric oxide synthase; ET-1 = endothelin 1; GC = glucocorticoid; IR = insulin resistance; FFA = free fatty acids; hs CRP = highly sensitive C-reactive protein; IGT = impaired glucose tolerance; IFG = impaired fasting glucose; LOAD = late-onset Alzheimer’s disease; MGC = microglial cell; Mɸ = macrophage; MC = mineralocorticoid; NO = nitric oxide; NALD = non-alcoholic liver disease; NASH = non-alcoholic steatohepatitis; O2- = superoxide; PCOS = polycystic ovary syndrome; T2DM = type 2 diabetes mellitus; RAAS = renin angiotensin aldosterone system; ROS = reactive oxygen species; TNFα = tumor necrosis alpha; XO = xanthine oxidase.

The current societal aging phenomenon as presented in Section 1.1 is associated with excesses and these excesses have been incorporated into a syndrome that the late Gerald M Reaven initially termed Syndrome X, which was later termed the now familiar metabolic syndrome (MetS) (Figure 2) [23]. MetS is a clustering or constellation of metabolic factors and clinical syndromes, which are intimately linked together by insulin resistance and known to increase the risk of not only cerebro-cardiovascular (CVD) disease and T2DM but also may link age-related LOAD. Importantly, visceral obesity is felt to be the initiating driver and insulin resistance is felt to be the linking factor of each of the four arms of the insulin resistance syndrome X (Figure 2). The ATP III guidelines provide the proper clinical parameters to identify those individuals with the MetS as follows: Three of the following five factors need to be confirmed: Central obesity (≥94 cm (for males), 80 cm (females)), triglycerides concentration ≥ 150 mg/dL, HDL cholesterol concentration < 40 mg/dL (males), < 50 mg/dL (females), values of blood pressure ≥ 130/85 mmHg and glycemia ≥ 100 mg/dL [24]. 

### 1.3. Insulin Resistance: Peripheral and Central-Brain Insulin Resistance 

**Scheme 1:**
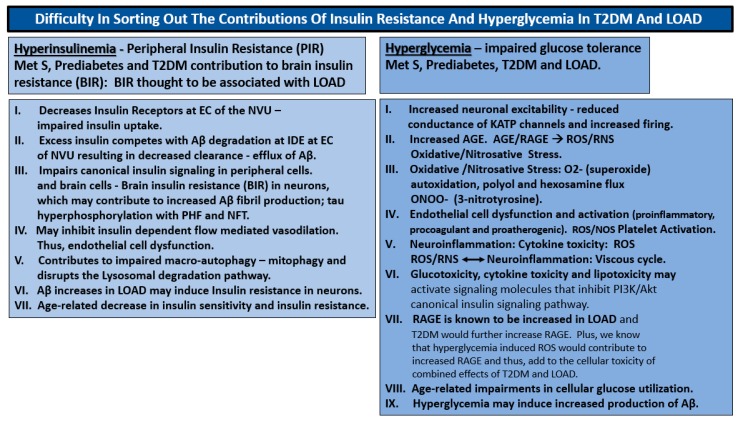
Difficulty in sorting out the contributions of insulin resistance and hyperglycemia in T2DM and late-onset Alzheimer’s disease (LOAD). Insulin resistance and hyperglycemia are both important in the development of the end-organ complications associated with T2DM including the brain. T2DM has both a singular early peripheral insulin resistance state that compensates and initially protects individuals from the development of hyperglycemia but not without a price to pay (due to the damaging effects of hyperinsulinemia) not only in peripheral tissues but also in the brain followed over time and aging with ongoing insulin resistance. Hyperglycemia eventually evolves due to the inability of the pancreatic islet beta cells to compensate (islet beta cell exhaustion-dysfunction and/or loss due to apoptosis) with ensuing hyperglycemia. This box attempts to outline what is currently thought to be known regarding insulin resistance and hyperinsulinemia and compare these effects to hyperglycemia in the brain that may contribute to the development of LOAD. Aβ = amyloid beta; AGE = advanced glycation end products; BIR = brain insulin resistance; EC = endothelial cells; IDE = insulin degrading enzyme; KATP = adenosine triphosphate-sensitive potassium channel; LOAD = late-onset Alzheimer’s disease; MetS = metabolic syndrome; NVU = neurovascular unit; O2- = superoxide; PHF = paired helical fragments; PIR = peripheral insulin resistance; NFT = neurofibrillary tangles; RAGE = receptor for advanced glycation end products; RNS = reactive nitrogen species; ROS = reactive oxygen species; T2DM = type 2 diabetes mellitus.

It is difficult to sort out the differences of peripheral insulin resistance (PIR) and hyperglycemia–glucotoxicity, since they are both present in T2DM and in brain insulin resistance (BIR) in LOAD (Scheme 1). 

Further, the effect PIR and glucotoxicity may have important bearings on the development of brain insulin resistance (BIR) in LOAD. For example, Ferreira LSS et al. utilize the concept that visceral omental obesity induces excessive toxic free fatty acids and hyperglycemia induces advanced glycation end products (AGE) to result in blood–brain barrier (BBB) permeabilization [25]. This was followed by brain neuroglia (astrocyte and microglia) inflammation with the production of a host of toxic cytokines and chemokines to induce neuronal BIR and endoplasmic reticulum stress with synaptic dysfunction and/or loss and their association with neuronal dysfunction and neurodegeneration in age-related LOAD. However, it is currently becoming more and more evident that there exists a definite primary BIR in LOAD [26,27,28,29,30]. 

### 1.4. Hypothalamic–Pituitary–Adrenal (HPA) Axis Dysfunction in the Metabolic Syndrome (MetS) 

The HPA axis in homeostatic conditions is a tightly regulated system that represents one of the body’s response mechanisms to acute and chronic physiological or psychological stress. HPA axis dysfunction is manifest in obesity, insulin resistance, MetS and T2DM with excesses of mineralocorticoids (aldosterone), glucocorticoids (corticosterone in rodents and cortisol in humans) and may be associated with increased sympathetic tone [9,31]. However, in diet induced obesity (DIO) Western models and preclinical diabetic *db/db* models and humans the HPA axis becomes dysfunctional. Elevated levels of aldosterone and corticosterone contribute to insulin resistance and cerebrocardiovascular disease and may even implicate the cerebral arteriole and capillary NVUs.

**Figure 3:**
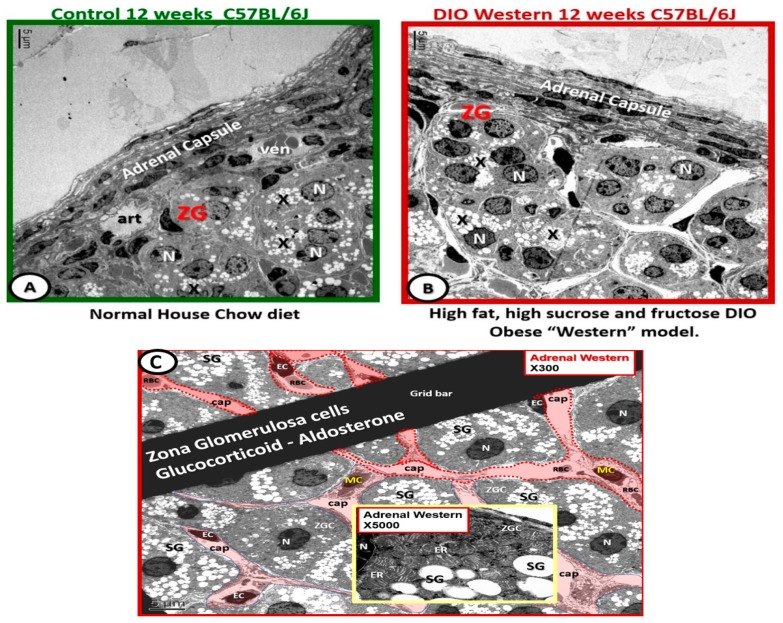
Adrenal gland zona glomerulosa hypertrophy and hyperplasia with capillary dilation in diet induced obesity Western models. Panel (**A**) illustrates the normal control zona glomerulosa (ZG). Panel (**B**) depicts the abnormal remodeling changes found in the ZG cells containing spherical secretory granules (electron lucent) that contain aldosterone (X). Note the hyperplasia and hypertrophy and dilated capillaries as compared to the control models in panel (**A**). Panel (**C**) depicts dilated capillaries bathing the zona glomerulosa cells in Western models. This image illustrates in greater detail the dilated capillaries (cap; pseudo-colored red) that appear to be continuously bathing the zona glomerulosa cells (ZGCs) and nuclei (N). Insert emphasizes the zona glomerulosa secretory granules (SG) and the extensive endoplasmic reticulum (ER). Magnification ×300; scale bar = 5 µm. Insert magnification ×5000; bar = 0.5 µm. EC = capillary endothelial cell(s); MC = mononuclear inflammatory cells; RBC = red blood cells. Magnification ×300; scale bar = 5 µm.

Previously, in DIO Western model cohorts [9] the adrenal gland underwent considerable ultrastructural remodeling (unreported data) (Figure 3). Importantly, the above abnormalities in the Western models strongly suggested the presence of inappropriate and impaired HPA feedback control with dysregulation between these neuroendocrine and systemic endocrine organs. These observations and findings in the Western model agreed with another study of the adrenal gland [32]. 

In addition to the HPA axis dysfunction it is thought that the visceral-omental adipose tissue excess is associated with increased compressive effects on the kidney that may result in an increase in the renin–angiotensin–aldosterone system (RAAS). Moreover, the excess aldosterone may also be associated with a visceral adipose-derived aldosterone releasing factor [33,34,35].

### 1.5. Type 2 Diabetes Mellitus, Late-Onset Alzheimer’s Disease and Mixed Dementias Intersections 

LOAD, like T2DM is a chronic age-related disease with a long preclinical or prodromal phase (up to 10 years), which is similar to prediabetes. Furthermore, in age groups over the age of 65 LOAD has a prevalence (10–30%) an incidence (1–3%) with a current lifetime risk of approximately 10.5% and may have a clinical duration of 8–10 years [36].

**Scheme 2:**
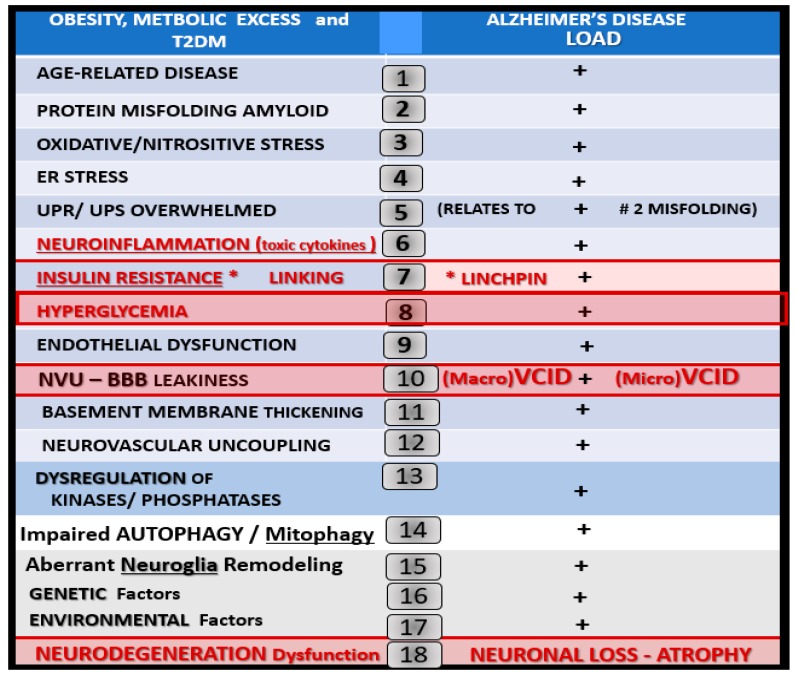
Multiple intersecting risks between T2DM and late-onset Alzheimer’s disease. This figure illustrates multiple (at least 18) intersecting risks between T2DM and LOAD. Further, these multiple intersects may contribute to the increased risk of LOAD in those individuals with T2DM. Importantly, note that insulin degrading enzyme (IDE) is related to #7 insulin resistance and hyperinsulinemia in that the elevated insulin of IR could compete with amyloid beta for degradation by the insulin degrading enzyme and that advanced glycation end products (AGE) and its receptor, the receptor for advanced glycation end products (RAGE), are important to #8 hyperglycemia in generating reactive oxygen species and AGE could also compete with RAGE for amyloid beta degradation. AD = Alzheimer’s disease; BBB = blood–brain barrier; ER = endoplasmic reticulum; LOAD = late-onset AD; NVU = neurovascular unit; T2DM = type 2 diabetes mellitus; UPR/UPS = unfolded protein response.

The MetS coupled with aging allow for multiple intersections between T2DM and LOAD with IR as the possible linking linchpin between these two age-related diseases (Scheme 2). 

The above converging global situations in Section 1.1 and Section 1.2 gives rise to great concern in that T2DM has recently been demonstrated to increase the risk of developing other chronic age-related diseases such as LOAD [37,38,39,40,41,42,43,44,45,46,47,48]. T2DM and LOAD are known to have multifactorial risk profiles and therefore, it is likely that the relationship between these two diseases have multifactorial intersecting risks that may promote cognitive abnormalities and interact during the development of LOAD with underlying T2DM. 

**Figure 4:**
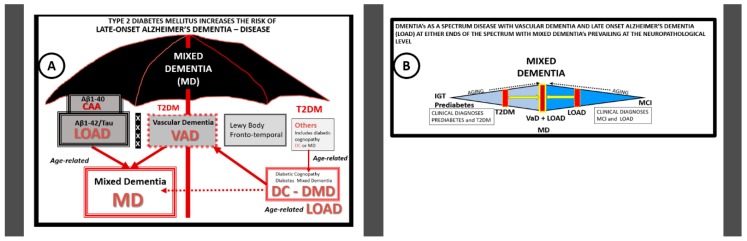
The overarching umbrella and the spectrum of mixed dementias in late-onset Alzheimer’s disease (LOAD). Panel (**A**) depicts the overarching umbrella of mixed dementia and diabetic mixed dementia in LOAD. LOAD is by far the most common of all dementias (90–95%) with early-onset Alzheimer’s disease contributing approximately 4–5%. What has been previously thought of as distinct dementias with rigid separating lines may now be thought to belong under the overarching umbrella of mixed dementia(s) (MD). Individuals who are clinically diagnosed with LOAD, to best of the clinician’s ability, during life, are frequently found have a mixed dementia at the time of autopsy and neuropathologic examination. The histopathology consists of not only amyloid beta extracellular neuritic plaques, microtubule associated tau paired helical fragments and neurofibrillary tangles but also cerebral amyloid angiopathy (CAA) and evidence of microvascular capillary neurovascular unit (NVU) and arteriolar abnormalities in structure and function. Interestingly, it is now being found that NVU dysfunction may precede the deposition of amyloid beta and tau in LOAD. Note the Xs within the thickened bar represents a softening of the hard-fixed boundary lines that previously divided LOAD from vascular dementia (VaD) and the other dementias under this overarching umbrella. The ‘others’ box in the figure (far right) may include the following: Metabolic, e.g., diabetic cognopathy or diabetic mixed dementia (DMD), trauma, infection, Parkinson’s disease, alcohol, rare genetic causes and drug-induced and depression-related causes of dementia. Panel (**B**) illustrates the spectrum of mixed dementias (MD) found in LOAD. LOAD and vascular dementia (VaD) are the two most common dementias in older individuals with LOAD being the most common. However, when brains are examined in those patients clinically diagnosed as LOAD it is found by autopsy and neuropathological examination that most have a mixed dementia (MD) of LOAD, cerebral amyloid angiopathy (CAA) and VaD making them mixed (co-occurrence) dementias (MD). Therefore, some now consider MDs as one of the most common forms of dementia in older adults aged 65 and older.

LOAD is responsible for 90–95% of all dementias. This contrasts with early onset familial Alzheimer’s disease, which develops prior to age 65 and may be related to specific genetic defects such as those in the amyloid precursor protein or presenilin genes and contributes to only approximately 4–5% of dementias [49]. Furthermore, there may be a continuum of progression from obesity, metabolic syndrome, T2DM to vascular dementia (VaD), LOAD and mixed dementia (MD). Importantly, there has been a recent trend to soften the once hard-fixed clinical and histopathologic boundary-lines drawn between vascular dementia (VaD) and LOAD. Additionally, LOAD may be considered to reside under the umbrella of mixed dementias (MD) (Figure 4). 

This fading phenomenon of the demarcation between VaD and LOAD is largely due to the findings of mixed dementia at the time of autopsy and the early finding of neurovascular–microvascular disease in LOAD (Figure 4) [50,51,52]. 

Moreover, recently recognized community-based neuropathologic studies have shown that there may be many different complex constellations of underlying pathologies, which may lead to cognitive decline in LOAD [53]. Mixed dementias equals co-occurrence dementias for example LOAD + VaD + CAA = mixed dementia when we include neuropathologic findings of post-mortem autopsy. Furthermore, it has been recently shared that neurovascular disease, particularly microvascular—NVU disease (vascular contributions of impaired cognition and dementia (VCID—microvascular, micro-VCID)—may be one of the earliest findings in LOAD [54,55]. Moreover, the two-hit vascular hypothesis has been placed into acceptance (in addition to other multiple LOAD hypotheses) [56,57] and recently the paper with 69 authors, which discussed in detail the importance of the NVU and its constituent cells as well as other related risk factors [58]. Microvascular NVU dysfunction, cellular remodeling and NVU uncoupling may result in hypoperfusion and decreased CBF (discussed in greater detail in Section 4.1), which appear to be early markers of neurodegeneration. These remodeling changes may set in motion a serious aberrant cascade of remodeling and functional events in the brain that may be concurrent or even precede the synaptic dysfunction and deposition of neuritic plaques or neurofibrillary tangles and result in the cognitive decline of age-related LOAD neurodegeneration. It is commonly known that T2DM increases the risk for VaD and a recent study of 28 prospective observational studies in 2012, which included 89,708 diabetic patients demonstrated a 73% increased risk of all type dementias (which included LOAD), a 56% increase of LOAD and a 127% increase of VaD in diabetic human patients [59]. However, it might be very interesting if one would consider LOAD as a mixed dementia (inclusive of VaD, CAA and LOAD) in regards to how these percentages might change. 

### 1.6. Continuum of Progression: From Obesity, Insulin Resistance to T2DM to LOAD 

**Figure 5:**
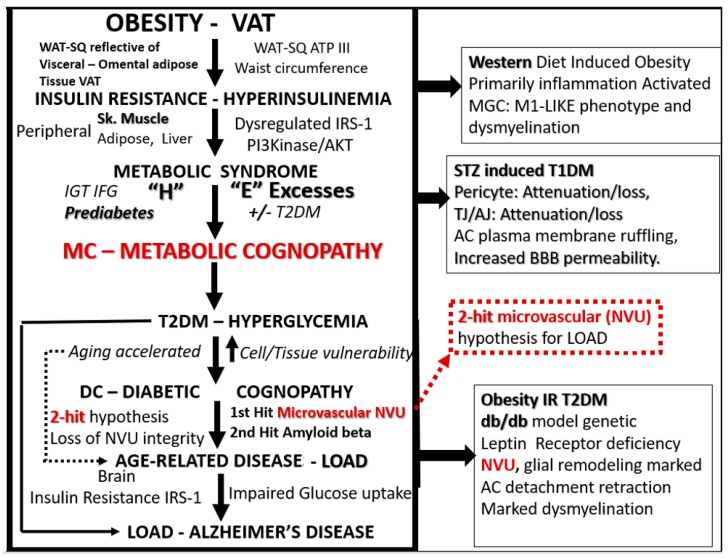
The progressive continuum from visceral obesity to type 2 diabetes mellitus to neurodegeneration and late-onset Alzheimer’s disease (LOAD). This continuum of progression (downward arrows) may occur independently as independent risk factors or interact synergistically and concurrently over time (aging). This figure also represents the continuum of progressive metabolic excesses and stresses involved to advance to LOAD via obesity, insulin resistance, metabolic syndrome and a metabolic cognopathy to prediabetes and/or overt T2DM with glucotoxicity to accelerated aging and increased neurovascular unit, vascular mural cells, neuroglia, neuronal cellular vulnerability and remodeling to metabolic cognopathy, diabetic cognopathy and aging with the increased risk of neurodegeneration and LOAD. This figure proposes that these above precursors are more of a continuum of progression from various stages, one to the next; however, some have posited that they could each represent separate pathologies that may be differentially expressed over time and thus remain age-related. The three boxes on the right side of this figure refer to our previous independent findings in the brains of the diet induced obesity, insulin resistant model with impaired glucose tolerance or prediabetes Western mouse model [9], the streptozotocin induced type 1 diabetic mouse model [10] and the *db/db* mouse model of obesity, insulin resistance and T2DM [5,6,7,8]. Note the intersects between #10 in Scheme 2 to the red dashed outline of the two-hit microvascular hypothesis and vascular contributions to cognitive impairment and dementia (VCID). ATP III = adult treatment panel III of the National Cholesterol Education program; cognopathy = disease of or impaired cognition; DC = diabetic cognopathy; “H” = the four hyper-conditions of the metabolic syndrome; IFG = impaired fasting glucose; IGT = impaired glucose tolerance; IRS-1 = insulin receptor substrate-1; MC = metabolic cognopathy; NVU = neurovascular unit; PI3kinase/AKT = phosphatidylinositol 3/protein kinase B; Sk = skeletal; SQ = subcutaneous; VAT = visceral adipose tissue; WAT = white adipose.

There appears to be a continuum of progression in the natural history of T2DM inclusive of obesity, MetS to LOAD. The following figure examines the progression from obesity (specifically visceral or omental) to MetS to T2DM and the multiple intersects and increasing cellular vulnerability to the development of LOAD and mixed dementia(s) (MD) (Figure 5). 

As mentioned earlier in Section 1.2, obesity seems to be the driver of the MetS. (Figure 2) with associated mild cognitive impairment (MCI) to T2DM to NVU remodeling, neuroinflammation, neurodegeneration and LOAD. Peripheral insulin resistance (PIR) and central brain insulin resistance (BIR) and impaired insulin signaling seem to be the link between T2DM and LOAD. So, it seems that T2DM may increase the vulnerability and the risk of LOAD via a progressive continuum: From visceral-omental mid-life overweight and obesity that is known to independently increase LOAD as well as VaD [60]. This progressive continuum to LOAD via the MetS to T2DM seems to increase the risk of neurodegeneration via a two-hit vascular hypothesis: VCID to LOAD (Figure 5). 

Previous studies in obese-prediabetic models with impaired glucose tolerance have demonstrated that the diet-induced obesity Western model (with high fat, high sucrose and fructose in C57B6 models) were observed to have lipofuscin-like bodies within the interstitial spaces that were being avidly consumed by activated microglial cells with some dysmyelination (markedly less electron density of myelin) of axons in layer III of the mid cortical grey matter. Overall, this model was primarily a neuroinflammatory model with activation and senescence of microglia without any NVU remodeling [9]. The next model was the lean streptozotocin induced type 1 diabetes model, which demonstrated marked NVU remodeling with blood–brain barrier (BBB) disruption and neurovascular unit dysfunction with increased permeability and loss of NVU BBB integrity. In this model we deduced that glucotoxicity resulting in excessive oxidative stress was the primary cause of NVU remodeling, since treatment with a specific mitochondria carbonic anhydrase inhibitor protected these NVU remodeling changes as well as increased permeability [10]. The most recent model studied was the obese, insulin resistant, T2DM female *db/db* mouse model [5,6,7,8]. This model demonstrated marked NVU, microglia cell(s) (MGC) and oligodendrocyte(s) (OL) and myelin remodeling that was protected utilizing a 10-week treatment period with the glucose lowering anti-diabetic sodium glucose transporter 2 (SGLT2) inhibitor from 10–20 weeks of age [8]. In this diabetic obese *db/db* model we were also able to demonstrate a marked expansion in aortic visceral perivascular adipose tissue that contained hypertrophic unilocular adipocytes that were rupturing with the spillage of neurotoxic proinflammatory free fatty acids (unpublished data). These remodeling changes were also associated with chronic inflammation due to peripheral systemic macrophage infiltration and thus provided for excessive neurotoxic cytokines and adipokines to be available to the aortic wall and systemic circulation, which was associated with vascular stiffening. This vascular stiffening could increase abnormal pulsatile mechanical forces associated with NVU—microvascular structural damage and remodeling to the NVU of brain due to increased pulse wave velocity and increase the expansion via increased pulse pressure in addition to also being a source of chronic inflammation due to systemic cytokines-adipokines from the perivascular adipose tissue. Thus, the *db/db* model [5,6,7,8] was able to ‘fill-in’ many of our gaps in knowledge between the diet-induced obesity Western model [9] and the type 1 streptozotocin induced diabetic models [10]. 

## 2. Capillary Neurovascular Unit (NVU) 

**Figure 6:**
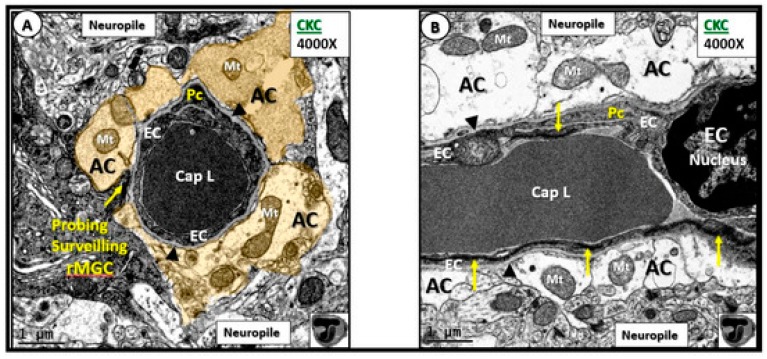
Normal neurovascular unit (NVU) morphology in control wild-type non-diabetic models (C57BL/KsJ; CKC control). Panels (**A**) (cross-section) and (**B**) (longitudinal section) illustrate the normal cellular ultrastructure of the NVU. Panel (**A**) depicts an electron dense ramified microglia cell (rMGC) surveilling the NVU (arrow). The NVU capillary consists of an endothelial cell (EC) encircling a capillary lumen (Cap L) whose basement membrane (BM) splits (arrowheads) to encompass the pericyte (Pc) foot process. Note how the pseudo-colored golden astrocyte (AC) end-feet encompass and tightly abut the capillary endothelial cell (EC) and pericyte (Pc) BMs. Note that the astrocyte (AC) clear zone in panel (**A**) was pseudo-colored golden to emphasize its importance in the NVU, while it exists as a clear-zone with a reduced electron dense cytoplasm as compared to other cells within the brain, and represents not only a golden halo, but also a clear zone or corona of AC foot processes surrounding the EC and Pc cells of the NVU (panel (**B**)). Panel (**B**) illustrates the electron-lucency of the AC clear zone halo or corona that tightly abuts and encircles the NVU EC and Pc BMs. Note the EC nucleus (far right side) and the highly electron dense tight junctions/adherens junctions (TJ/AJ) complex (yellow arrows) that are not readily visible in panel (**A**) (arrows). Moreover, note that the mitochondria (Mt) have an electron dense Mt matrix and that cristae may be noted even at this magnification. Note that the NVU is encompassed by the outermost abluminal neuropil (neuropil). In the bottom right-hand corner, note the logo of red blood cells within a capillary NVU that are in the shape of the letter T overlying the letter J, which are used to abbreviate tight junction(s). Magnification ×4000; scale bar = 1 µm. CC by 4.0 [5].

The capillary NVU is a readily identifiable ultrastructural constant when viewing brain tissue with transmission electron microscopy (TEM) (Figure 6) [5,6,7,8]. 

This multicellular structural and functional unit in the brain consists of the following cells: ECs, pericyte(s) (Pc), astrocyte(s) (AC), MGCs, OLs and neurons. Within the NVU is contained the EC paracellular BBB synthesized primarily by ECs and supported structurally and functionally by Pcs and additionally supported and maintained by a corona of surrounding–encircling AC foot processes (ACfp). The two primary mural cells of the capillary NVU are the luminal continuous monolayer of ECs and supportive Pcs, which are embedded within the EC basement membrane (BM) creating an inner and outer BM. This contrasts with the arterial NVU, in which vascular smooth muscle cells provide for mural arteriole support with similar abutting of ACfp. The third contiguous cells belong to the multiple ACfps that tightly abut the EC and Pc outer BMs. Probing ramified microglia cells, oligodendrocytes and oligodendrocyte linage precursor cell(s) (OPC) (especially in subcortical and white matter regions) along with neuronal axons (unmyelinated and myelinated) may also be noted to be adjacent to the outer basement membrane of the NVU [5,6,7,8]. 

There are basically three to four barriers of the NVU from luminal to abluminal regions: i. Endothelial glycocalyx (ecGCx); ii. endothelium (ECs) inclusive of BBB tight and adherens junctions (TJ/AJ) and the EC cytoplasm itself, which also utilizes transcytosis as a method of EC cellular transport; iii. EC and pericyte (Pc) and/or its pericyte foot processes (Pcfp) basement membrane (BM), which shares the common inner and outer basement membrane (BM); iv. astrocytes (ACfp) and their outer basal lamina. Of these three to four barriers, the BBB TJ/AJ that reside between EC cell–cell junctions of the NVU and between the blood and brain interstitial fluid provides the greatest trans-endothelial electrical resistance (TEER) control over the immediate microenvironment of brain cells. Additionally, there are other interfaces–barriers that are also important such as the choroid plexus epithelium between the blood and ventricular cerebrospinal fluid (CSF), and the arachnoid epithelium between the blood and subarachnoid CSF. 

It is important to note before proceeding that the multicellular maladaptive ultrastructural remodeling figures shared in the review may represent a unique phenotype in the female leptin deficient *db*/*db* models with elevations in leptin and may not be a general phenotype in multiple other diabetic preclinical models; however, these findings seem to correlate with other ultrastructure studies of the NVU and neuropil in preclinical rodent models. 

### 2.1. Endothelial Cell(s) (EC)—Endothelium 

**Figure 7:**
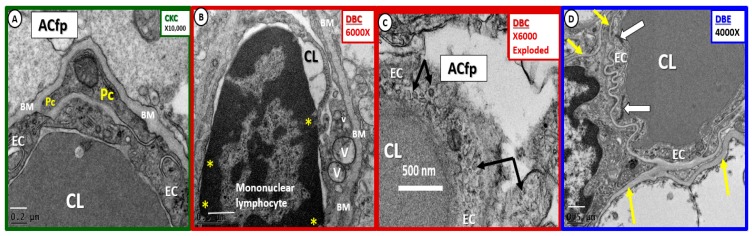
Multiple endothelial cell remodeling changes in diabetic *db/db* models. Panel (**A**) Illustrates the normal endothelial cell (EC), pericyte (Pc) and astrocyte foot process (ACfp). Note that the basement membrane appears almost as thick as in the known BM thickening in panel B; however, note that the magnification is higher (×10,000) and the scale bar is not as wide in panel A (200 nm) as in panel B (500 nm). Panel (**B**) depicts adherent mononuclear lymphocyte. Moreover, note EC vacuoles (V), vesicles (v) and pinocytosis arrows in panel (**C**). Panel (**D**) demonstrates the protective quality of empagliflozin treatment. Magnification ×10,000 panel A; ×6000 panel B. ×6000; bar = 500 nm panel C; ×4000 panel D with scale bars 0.2 µm, 0.5 µm, 0.5 µm in panels A, B, D, respectively. CC by 4.0 [5].

The endothelium is the continuous, epithelial, highly polarized, monolayer cell that lines the luminal surface of the NVU and serves as the interface between the peripheral circulating blood and its cellular, molecular proteins, ion constituents and the brain parenchyma (Figure 7). The NVU ECs are responsible for creating and maintaining the NVU permeability and lumen formation [61]. The EC is responsible for 2 initial barriers, which create the BBB consisting of tight and adherens junctions (TJ/AJ), junctional adherens molecules and cytoskeletal zona-occludin-1 and beta catenins that are formed between adjacent endothelial cells [5,6,7,8] and the EC surface layer the endothelial glycocalyx (ecGCx). The apical luminal and basolateral abluminal polarity of the brain EC is of utmost importance in creating the normal requisite physiologic functions of the NVU in that many of its influx–efflux and specific transporter proteins require a specific location to be physiologically functional [5,61]. It is also important to note that while the integrity of the paracellular TJ/AJ proteins in ECs are of critical importance in EC permeability, there also exists an intracellular transcytotic route for increased EC transcellular transport and permeability mechanisms. In fact, our diabetic *db/db* models have demonstrated that there exists not only an ultrastructural abnormality in TJ/AJ with attenuation and/or loss but also an increase in the EC transcytotic-pinocytotic vesicles plus other important remodeling change such as inflammation and basement membrane thickening [5,8] (Figure 7). 

Moreover, the EC in the diabetic db/db model demonstrated the following remodeling changes: i. Endothelial thinning and loss of electron density; ii. increased vesicles-pinocytosis-vacuoles; iii. aberrant mitochondria; iv. hyperplasia with some reduplication; v. endothelial cell activation with adherent erythrocytes, leukocytes (lymphocytes and monocytes) and platelets. Importantly, the EC is capable of synthesizing at least two main autocrine paracrine vasodilating molecules nitric oxide (NO) and prostaglandin E2 (PGE2) or prostacyclin and the autocrine paracrine potent vasoconstrictor endothelin-1 (ET-1). Recently, normal aging in Wistar rats increased EC permeability in 14–16-month-old cohorts as compared to younger cohorts at 2–3 months of age [62]. Bors L. et al. were able to demonstrate that there was increased thickness of EC BM, TJ/AJ were attenuated, ACfp were extended with increased glial fibrillary acidic protein (GFAP) staining and a decrease in the efflux permeability glycoprotein (P-gp) or the ABCB-1 efflux EC protein [62]. The endothelium is responsible for synthesizing its own luminal endothelial glycocalyx and the inner BM that are continuous with the Pc assisted synthesis and maintenance of the inner and outer BMs of the NVU. 

### 2.2. Endothelial Glycocalyx (ecGCx) 

**Figure 8:**
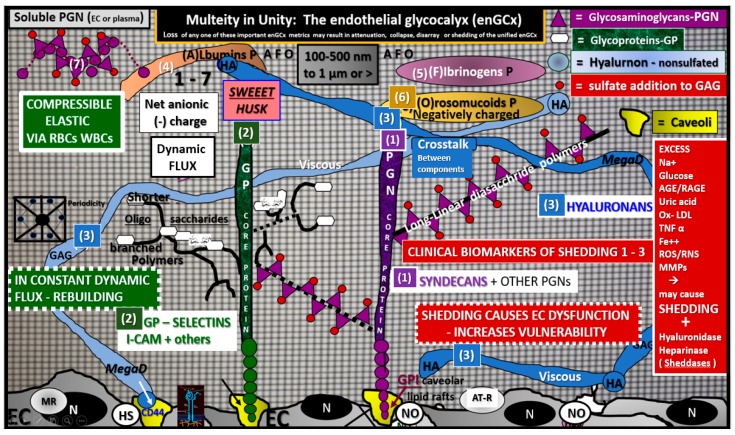
The endothelial cell glycocalyx (ecGCx) of the capillary neurovascular unit. This cartoon depicts the three major anchoring proteins of the ecGCx: Proteoglycan(s) (PGN) (1) (purple), glycoproteins (GP) (2) (green) and hyaluronan (HA) (3) (blue). This ecGCx sweet husk of the endothelial cell (EC) is in a constant state of flux being constantly resynthesized by the EC and is highly compressible by the blood’s cellular constituents (red and white blood cells—RBC and WBC). The proteoglycans (PGN) are noted for their syndecans (SYN) (plus others) and the glycoproteins (GP) are known for their selectins such as I-CAM, P and E-Selectin plus others. One will note the glycosaminoglycans (GAGs) long-linear disaccharides of the PGNs and shorter highly branched GAGs oligosaccharides of the glycoproteins. This surface layer has a net negative anionic charge (due to orosomucoids protein and opposes negatively charged molecules in the blood utilizing Van der Wall forces) and size selective semipermeable surface barrier (hindering access of 70 kiloDalton- kDa or larger proteins) and additionally serves as a glucose and sodium sink initially in health that can be rapidly overcome with attenuation and/or shedding in diabetes (glucotoxicity–oxidative stress) and clinical excesses of sodium chloride salinity. The intact ecGCx contains the protective extracellular superoxide dismutase (ec-SOD) and antithrombin III (AT III) while serving as a mechanotransducer. The thickness of the ecGCx varies from 100–500 nm (300 nm mean), from 1–2 micrometers in length and the percentage of EC covered by the ecGCx was approximately 40% in the NVU capillaries in the transmission electron microscopic studies and was greater in the brain as compared to the heart or lung continuous capillaries without a BBB (Figure 9) [63]. Glucotoxicity, oxidative stress and neuroinflammation (as in the diabetic *db/db* model), traumatic shock injuries, ischemia/reperfusion and sepsis are known to be capable of shedding the ecGCx and/or a reduction in its volume and plasma levels of syndecans, I-CAMs, selectins and hyaluronans can be measured clinically with elevated levels reflecting the reduced ecGCx due to thinning, loss or shedding. AT-1R = angiotensin 1 receptor; BBB = blood–brain barrier; CD44 = the hyaluronan receptor of the ecGCx and cell-surface glycoprotein involved in cell–cell interactions and binds HA via the amino-terminal domain; EC = endothelial cell; GP = glycoprotein(s); H2S = hydrogen sulfide; HA = hyaluronan; I-CAM = intercellular adhesion molecule 1; MR = mineralocorticoid receptor; N = nucleus; P = protein; PGN = proteoglycans; RBC = red blood cell; WBC = white blood cell.

The ecGCx is a decorative ‘coat of many colors’ and consists of a sugar-protein endothelial screen-like mesh; gel-like slime surface coating that covers and decorates the luminal side of the highly polarized endothelium and is synthesized primarily by the EC with some plasma contributions of albumin, fibrinogen and soluble plasma proteoglycans and glycolipids (Figure 8). 

The intact ecGCx is important for vascular wall arteries, arterioles and capillary integrity and it does not decorate veins or venules [64]. The ecGCx is anchored to the EC via proteoglycans (syndecans plus others) and glycoproteins (selectins plus others including I-CAM), while the third major component protein consists of hyaluronan. Hyaluronans may be free or anchored to the endothelium via CD44. Interestingly, this protective coating is very similar to the brain’s interstitial extracellular matrix. The elusive ecGCx is in a constant state of flux of being regenerated and repaired by the endothelium and plasma constituents [65,66,67,68]. The systemic cells and molecules of the blood seldom see or come into direct contact with the plasma membrane-plasmalemma of the EC, because it is constantly and nearly continuously covered by the protective ecGCx surface coating, which prevents direct exposure of systemic blood components to the EC plasma membrane in health. 

**Figure 9:**
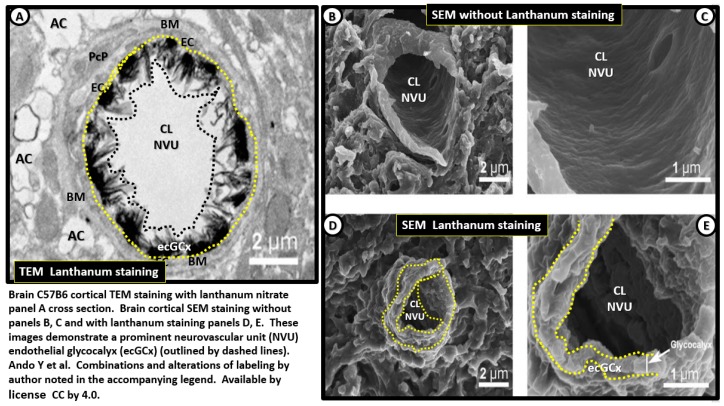
TEM and scanning electron micrographs of the endothelial cell glycocalyx, with and without lanthanum staining. Panel (**A**) depicts a transmission electron microscopic (TEM) cross sectional image of the endothelial cell glycocalyx (ecGCx) of the neurovascular unit, outlined by yellow dashed lines abluminally and black dashed line luminally. Note how this electron dense stained ecGCx by lanthanum nitrate with perfusion fixation covers nearly the entire luminal surface of the endothelial cell (EC) with intermittent varying electron dense cores and extend outwardly into the lumen in control non-diseased models of the C57B6 mice from the cerebral cortical brain. Scale bar = 2 µm. Panels (**B**) and (**C**) illustrate scanning electron microscopy (SEM) micrographs the NVU without lanthanum nitrate staining and panels (**D**) and (**E**) depict lanthanum nitrate staining and now, note the visible elusive ecGCx outlined by yellow dashed lines from the EC on the luminal side. This image actually combines Figure 1 and Figure 2 from Ando Y [63]. Scale bar = 2 µm Panels A, B and D. Scale bar = 1 µm panels C, E. CC by 4.0 [63].

Recently, the brain’s NVU ecGCx has been beautifully studied, visualized and documented by lanthanum nitrate staining by both transmission electron microcopy (TEM) and scanning electron microscopy (SEM) making this EC surface coating layer visible even though TEM preparations undergo numerous dehydration steps [63]. The perfusion fixation with lanthanum nitrate pre-sacrifice allows the ecGCx to be beautifully stained and visualized with TEM and SEM studies from cortical brain regions of the C57B6 mice (Figure 9). 

**Figure 10:**
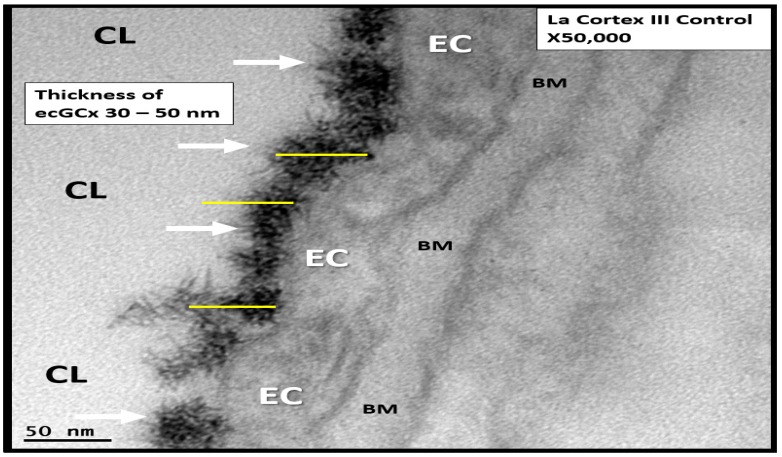
Lanthanum nitrate perfusion fixation staining of control C57B6 mouse models in the cortical layer III capillary neurovascular unit endothelial cell glycocalyx (ecGCx). Note the extreme electron dense staining of lanthanum nitrate covering the endothelial surface of the ecGCx (arrows) similar to Figures 12 and 13 that notably does not penetrate the endothelium or any other aspects of the neurovascular unit with lanthanum nitrate staining. Its thickness appears to vary from 30 to 50 nanometers (nm). Yellow scale bars of 50 nm are placed overlying the electron dense lanthanum nitrate staining ecGCx. Magnification ×50,000; scale bar = 50 nm. BM = endothelial cell basement membrane; CL = capillary lumen; EC = endothelial cell cytoplasm.

Moreover, our TEM core has recently been able to identify the elusive ecGCX with perfusion fixation by lanthanum nitrate in the cerebral NVU capillaries of control C57B6 mouse models and it will be interesting to observe for attenuation/thinning, shedding or degradation of this endothelial surface coating in age-matched diabetic models in the coming future (Figure 10). 

Aging is known to be the major risk factor for the development of LOAD, and it has recently been demonstrated that aging results in a thinning of the ecGCx by 50% when comparing young (6 months) to old (24 months) C57B6 mice (mesenteric and skeletal microvessels) and 30% thinning of the ecGCx when comparing young (23 years) and old (60 ± 2 years) humans (sublingual microvessels) [69]. Now that we may have a reliable and reproducible method for determining ecGCx disruption, thinning, degradation and/or shedding we may be able to demonstrate by TEM with perfusion fixation staining with lanthanum nitrate that indeed there is disruption of the endothelial surface layer—ecGCx—and in some cases be able to determine if the increased permeability that was once thought to be non-disruptive may now be considered to be disruptive due to alterations of the ecGCx [70]. 

### 2.3. Endothelial Cell Basement Membrane (BM) 

**Figure 11:**
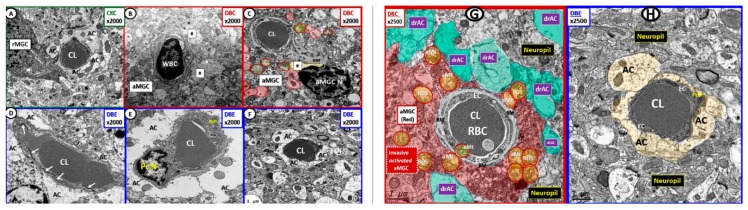
Empagliflozin protects the neurovascular unit from basement membrane thickening: Loss of blood–brain barrier endothelial tight and adherens junctions and pericytes. Panels (**A**) and (**D**) depict the normal ultrastructure of the basement membrane (BM) and BBB in control non-diabetic CKC models. Panels (**B**) and (**C**) depict the Pc ghost (panel B) and complete loss of Pcs in panels B and C as well as the detachment of astrocyte foot processes in panel A (hashtags) with invading activated microglia cell (aMGC) in the *db/db* DBC diabetic models outlined in red. Panels (**D**–**F**) demonstrate that empagliflozin protects from EC activation, loss of EC tight and adherent junctions (arrows), Pc loss and AC detachment in the diabetic *db/db* (DBC) treated models with empagliflozin (DBE) outlined in blue. Magnification ×2000; scale bar = 1 µm. AC = astrocyte end feet processes; aMGC = activated microglial cell; CL = capillary lumen; Pc N = pericyte nucleus; PcP = pericyte process; rMGC = ramified microglia cell. Image available via license CC by 4.0 [5,8]. Panels (**G**) and (**H**) depict the thickening of the BM in *db/db* models and the normal appearing BM in empagliflozin treated models, respectively. Note the invasive aMGC and the detached retracted ACfp in addition to the basement membrane thickening in panel G as compared to the empagliflozin treated models in panel H. Magnification. ×2500; scale bar = 1 µm. aMGC = activated microglia cell; aMt = aberrant mitochondria; drAC = detached retracted AC; EC = endothelial cell; CL = NVU capillary lumen; Pc = pericyte foot process; RBC = red blood cell; ROS = reactive oxygen species. CC by 4.0 [5,6,8].

The BM consists of type IV collagen, fibronectin, laminin, nidogen, and heparin sulfate proteoglycans (agrin and perlecan) and envelops the basilar portion of the EC and splits to encompass the Pc and forms its base to which the EC and Pc BM forms the adhesion anchoring structure for the corona of ACfps [5,71]. The BM is synthesized primarily by the EC; however, the Pc and AC also contribute to the synthesis and maintenance of the NVU BM. Interestingly, as one studies the ultrastructure of the NVU, one notes that polarized ECs create a barrier at the luminal surface layer (ecGCx), the paracellular and basilar abluminal side of the EC’s monolayer. In diabetic *db/db* models there is BM thickening and even though it is thickened, its structure is remodeled with rearrangement and thickening, which allows for increased permeability (Figure 11). 

### 2.4. Neurovascular Unit (NVU) Pericyte(s) (Pc) 

**Figure 12:**
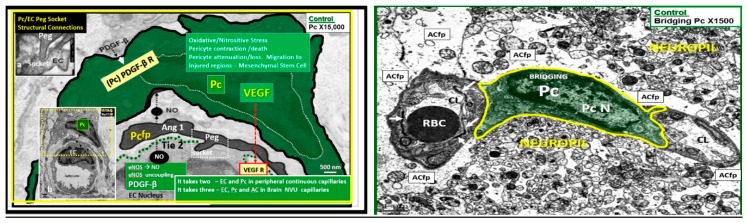
Normal pericyte–endothelial morphology and crosstalk in control CKC models. Left panel depicts a peripheral continuous capillary and demonstrates that “it takes two” in order to provide crosstalk homeostasis. Indeed, for both peripheral and brain continuous capillaries of the neurovascular unit (NVU with blood–brain barrier (BBB) tight and adherens junction (TJ/AJ)) it takes these two highly integrated vascular mural cells (endothelial cells (EC) and pericyte (Pc) interaction and crosstalk). However, in the brain “it takes three” since ECs, PCs and astrocytes (ACs) must properly interact in order for the proper development and maintenance of the BBB TJ/AJ-junctional adherens and zona-occludin-1 (ZO-1) as well as the glial astrocyte (AC) to allow for this microvascular system to develop proper cerebral blood flow, prevent increased permeability, and assure proper NVU coupling with regional neurons to assure proper functional hyperemia. This image focuses on peripheral continuous capillaries in order to demonstrate normal EC-Pc morphology and crosstalk. The Pericyte soma is pseudo-colored green and its nucleus is enveloped with a white dashed line. Importantly the Pc stains positive for alpha smooth muscle actin and allows for contraction and importantly relaxation to allow for NVU coupling and vasodilation. Insert (a) depicts the peg socket interaction while insert (b) allows the entire capillary to be viewed in its environment without colorization. Magnification ×15,000; bar = 500 nm in all images. Moreover, note that the EC provides nitric oxide (NO) and platelet-derived growth factor (PDGFβ) and that the Pc provides vascular endothelial growth factor (VEGF) and makes possible the contraction and relaxation of the capillary NVU via Pc actin which is essential for these two cells’ survival and the Ang1/Ti2 system via crosstalk for proper function and protection of the endothelial capillary NVU of control CKC models. Right panel Illustrates the pericyte connectivity between two capillary neurovascular units in cerebral cortex grey matter of the C57B6 mouse control model layer III. ACfp = astrocyte foot processes; arrows = tight and adherens junctions; CL = capillary lumen; eNOS = endothelial nitric oxide; NVU = neurovascular coupling; PDGF-β = platelet-derived growth factor; PDGF-R its receptor; VEGF = vascular-derived growth factor and VEGF receptors—VEGF-2 (Flk-1/KDR = type III receptor tyrosine kinase); Pc = pericyte; PcN = pericyte nucleus.

The pericyte (Pc) is a ubiquitous–systemic (found both in peripheral and brain microvascular continuous capillaries), requisite, mesenchymal-derived, pluripotent and postnatally undifferentiated vascular mural cell important for mediating physiological and pathological repair processes. The Pc serves other microcirculation functions including post-natal vascular development (angiogenesis), important for maturation and remodeling of the NVU. Additionally, pericytes provide microvascular structural stabilization as well as a supportive–protective role to capillary endothelial cells and is known to be an innate immune and antigen presenting cell of the NVU, which may be capable of differentiating into a microvascular niche mesenchymal stem cell (Figure 12) [72,73,74,75,76,77,78,79,80,81]. 

Importantly, pericytes are contractile cells and contribute to the regulation of capillary cerebral blood flow, hydrostatic balance and maintenance of proper intracapillary pressure and permeability between the microvascular NVU and interstitial tissue [74,75,76,77,78,79,80]. The EC provides the Pc with essential NO and platelet-derived growth factor beta (PDGFβ) to allow for prevention of contraction and allows for dilation of the capillary NVU–capillary neurovascular unit maintenance. Moreover, the EC relies on the Pc to provide VEGF production (essential cellular crosstalk). While each of these above interactions between pericytes and endothelial cells are extremely important it is beyond the scope of this review to discuss them in their entirety, especially in wound healing and angiogenesis.

Previously, some have considered the Pc to be the “guardian angel” or sentinel of the EC microcirculation within the peripheral continuous capillaries (Figure 12) [62,73,81,82,83]. This same “guardian angel” analogy may also be applied to the EC of capillary NVU as a functional and structural unit in the brain, since platelet-derived growth factor beta receptor (PDGFR-***β)*** knockout models in Pcs have demonstrated the abnormal formation of the NVU in adult models with increased permeability due to impaired BBB TJ/AJ development and function and impaired clearance of Aβ [74,75,76,77,78,79,80]. Additionally, its very important role within the NVU has undergone considerable expansion regarding its role in the effect of the regional neuronal activity of the neurons via the connecting astrocyte to result effectively in dilation in order for NVU coupling and functional hyperemia to support the necessary vasodilating properties of the capillary NVU and the smallest of arterioles [5,8,74,75,76,77,78,79,80]. 

**Figure 13:**
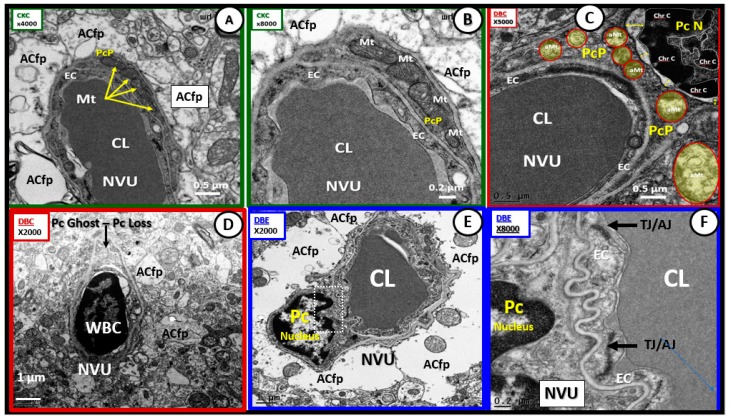
Pericyte injury and loss in diabetic female *db/db* with protection by empagliflozin. Panels (**A**) and (**B**) demonstrate the normal appearance of pericyte (Pc) and foot processes (Pcfp) in relation to the neurovascular unit (NVU) endothelial cell (EC) in control CKC models. Magnification ×4000; bar = 0.5 µm in A and ×8000; bar = 0.2 µm in B. Panel (**C**) illustrates injury-abnormalities (aberrant mitochondria and Pc Nuclear retraction and increased chromatin condensation) in diabetic DBC outlined in red. Magnification ×3000; scale bar = 0.5 µm. Panel (**D**) illustrates an activated endothelial cell with white blood cell adherence (lymphocyte) with loss of pericyte and foot processes in diabetic DBC outlined in red. Magnification ×1000; bar = 0.1 µm ghost cell. Panels (**E**) and (**F**) depict the protection of the Pc and Pcfp in the empagliflozin treated modes (DBE). Magnification ×2000; bar = 1 µm (panel E) and magnification ×8000; bar = 0.2 µm (panel F). CC by 4.0 [5].

Recently, it has been shared that pericytes (Pc) in the female diabetic *db/db* model undergoes marked remodeling changes including attenuation, retraction and/or complete loss from the cortical layer III NVU. These remodeling changes have also been demonstrated to be protected with empagliflozin treatment for 10 weeks duration from 10 to 20 weeks of age (Figure 13) [5,8].

### 2.5. Astrocyte (AC)–Diabetic Gliopathy

In the type 2 diabetic *db/db* model, I have put forth the term “diabetic gliopathy”—impaired glia function and ultrastructure in order to describe the dysfunctional and maladaptive ultrastructural remodeling response due to the injuries imposed upon glia cells (astrocyte 2.5., microglia 2.6. and oligodendrocyte 2.7.) as a result of obesity, insulin resistance–hyperinsulinemia and diabetic hyperglycemia–glucotoxicity of the *db/db* models [5,6,7] along with the multiple intersections between T2DM and LOAD—especially oxidative/nitrosative stress—in Section 2 and Scheme 2.

**Figure 14:**
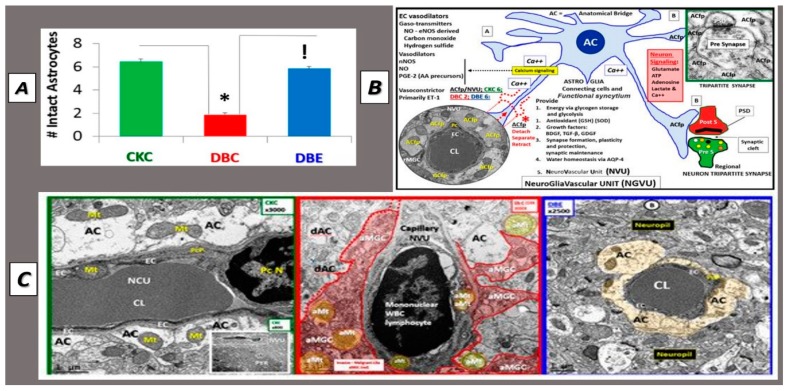
Detached Retracted Astrocytes in *db/db* Models. Panel (**A**) depicts the reduction in the numbers of intact–attached astrocyte foot processes in the diabetic *db/db* models and protection due to empagliflozin treatment. Panel (**B**) illustrates the normal relationship between the NVU astrocytes and neuronal synapses Panel (**B**). Panel (**C**) demonstrates the detachment and retraction of astrocyte foot processes (ACfp) (middle panel) as compared to the left-hand panel in control models and the protection of ACfp loss or detachment in empagliflozin treated models (right-hand panel). CC by 4.0 [92].

ACs are unique cells localized to only the brain and spinal cord tissues and they are the major strategic connecting cell within the brain. The astrocytic end-feet–foot processes (ACfp) of the capillary NVU are tightly adherent to the BM of the EC and Pc cells and play a specialized role for water, amino acid and ionic homeostasis; provide a reservoir for glucose storage via glycogen and are the primary source within the brain for localized antioxidant production of glutathione in addition to communicating with adjacent AC and bidirectionally with regional neurons for functional hyperemia–NVU coupling and cerebral blood flow (Figure 7, Figure 11, Figure 13 and Figure 14) [5,84,85,86,87,88,89,90]. Importantly, AC foot processes (ACfp) connect regional neurons to the mural cells of the NVU and are responsible for signaling the mural cells via glutamate signals, increases in calcium transients (Ca++ flux) from the neurons to the mural cell pericyte to dilate in the presence of increased neuronal activity and create the capillary NVU coupling which results in functional hyperemia and CBF. ACs are also connective in that they form a syncytium amongst other AC, which are in a constant state of sending or receiving information from other like AC cells. While it is a well-known and accepted theory that active neurons increase their nutrient-energy supply and oxygen by dilating nearby arterioles and capillary NVUs to provide for neurovascular coupling or functional hyperemia there does remain somewhat of controversy regarding whether it is the arteriole or capillary that play the major role. It is the opinion of the author that even though the capillary NVU may be the first to dilate that both capillary and arterioles of the brains vascular supply are affected. To this end, Mishra A et al. have recently demonstrated that AC’s signal pericytes but not the vascular smooth muscle cells of arterioles [91]. 

It is important to note that in *db/db* models that ACfp of the capillary NVU were detached, separated and retracted (Figure 7B, Figure 11, Figure 13B and Figure 14) [92]. Importantly, this would result in the loss of the connecting ACs from regional neurons to the capillary NVU and impair functional hyperemia [5,8,92] and CBF. These ultrastructural changes in the preclinical rodent *db/db* models if found to be present in human brain tissues could impair functional hyperemia and result in decreased CBF and contribute to a loss of energy substrate sources and decrease in oxygen resulting in ischemia and premature neuronal dysfunction in synapses and eventually dysfunction with known cognitive impairment and neurodegeneration. This detachment may be due to the increased endothelial, pericyte and AC oxidative stress generating excessive ROS/RNS due to glucotoxicity as previously proposed [5,8,91,92,93], which was protected with a SGLT2 inhibitor (empagliflozin) (Figure 14) [8]. 

### 2.6. Microglial Cell(s) (MGC), Neuroinflammation and Diabetic Gliopathy 

**Figure 15:**
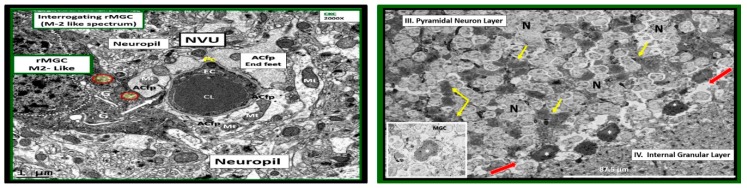
Ramified microglia interrogating neurovascular unit and pyramidal neurons layer III. Left panel illustrates an electron dense ramified microglial cell (rMGC) interrogating the neurovascular unit (NVU) in C57B6 control models (CKC). Note the rMGC protruding its cytoplasmic process and inserting between the astrocyte foot processes (ACfp) (arrow) or end-feet (~50–100 nm space) that encompass the capillary endothelium (EC) surrounding the capillary lumen (CL). Moreover, note the prominent Golgi system within the rMGC cytoplasm (G). Even in the rMGC there will be occasional aberrant mitochondria (pseudo-colored yellow with red outline) (Mt). Magnification ×2000; bar = 1 µm. CC by 4.0 [5]. Right-hand image depicts rMGCs with electron dense cytoplasmic extensions (yellow arrows) intermixed with pyramidal layer III neurons (N) of the cortical grey matter in control CKC 16-week-old mice. Note what we term a train of rMGCs with remodeled lipid bodies to form lipid cores (asterisks) in the interstitium between layers III and IV (red arrows). Inset is an exploded—highlighted image of the MGC with lipid core (asterisk). Scale bar = ~87.5 micrometer. Image information from focused ion beam/scanning electron microscopy (FIB/SEM technology): Voltage: 3 kV; pixel size: 350 nm; field of view: 175 × 175 µm^2^; image size 491 × 497; vacuum: 0.25 Torr [6].

MGCs are currently thought to be yolk sac-derived (mesoderm/mesenchymal), colonize the brain prenatally (embryonic day 10–14 in the mouse and week 4–24 in human brain), the innate first line of resident immune cell defense and thus, the immune guardians or gatekeepers of the brain [94,95]. MGCs may be considered the resident immunocompentent and phagocytic cell of the CNS and are critical to its normal functioning in health and homeostasis. Interestingly, they precede the appearance of the NVU, astrogliogenesis, oligodendrogenesis, neurogenesis, migration and myelination and are known to be regionally distributed. Ramified MGCs are constantly at work as a consummate gardener to provide a cleaning-housekeeping function and provide surveillance for waste clean-up from normal wear and tear of the brain’s cellular functional milieu. Their ramified processes are constantly moving about the brain ready to identify and phagocytose any unwanted accumulation of structural by products of metabolism in homeostasis or in response to injury mechanism [6,96]. In focused ion beam/scanning electron microscopic (FIB/SEM) videos one can even observe the rMGC cytoplasmic extensions as they appear moving about between neurons and the NVUs providing these important homeostatic housekeeping functions (Figure 15) [6]. 

MGCs have large numbers of membranous/intracellular microglial markers and a large number of signaling molecules, which include numerous microglia, cytokines and chemokines [97]. 

Additionally, they contribute to the regulation of brain development, shaping synaptic connectivity within neuronal networks and are of major importance in brain defense injury [6,97,98].

MGCs are readily capable of producing large amounts of free radicals (superoxide, reduced nicotinamide adenine dinucleotide phosphate oxidase (NADPH Ox), inducible nitric oxide synthase (iNOS) and mitochondrial-derived ROS) and are the major killing–phagocytic cell for infectious processes in the brain. Damage/danger-associated molecular patterns (DAMPs) or pathogen-associated molecular patterns (PAMPs) and MGC remain in an activated state until the DAMPS/PAMPs signal subside. Importantly, microglia are able to return to their surveilling-ramified phenotypes once the danger–damage signals or infectious invaders have been eradicated and assume their normal cellular debris housekeeping role of rMGCs [6,94,95,96,97,98]. While MGCs are essential to homeostasis they may also become injurious to the CNS cells as in the invasive damaging role they play in NVU BBB loss of integrity in diabetic models as a result of their invasiveness that is associated with AC detachment and retraction previously discussed in Section 2.5 [5]. 

rMGCs are genetically programed to constantly be prepared to undergo a rapid diverse phenotypic remodeling functional change to what may be termed activated amoeboid microglial cell phenotype (aMGC). These changes may be due to morphological remodeling and/or the expressions of their cell surface receptors in response to danger or damage signals such as PAMPs or DAMPs due to oxidized/glycated proteins/polypeptides, lipids, and nucleic acids from their diabetic hyperglycemic microenvironment.

aMGC have been classified by some to be similar to peripheral macrophages, i.e., M1-like (classically activated macrophages) and M2-like (alternatively activated macrophages) cells [99,100]. However, the possibly more preferred method of identification of MGCs relies on individual cell surface markers or their response to inducible cytokines and/or neurotoxins such as LPS (lipopolysaccharide) [101,102]. The author has chosen to utilize only the terms ramified (rMGC) or activated (aMGC) when referring to the morphofunctional–pathomorphologic phenotypic polarization in TEM observations [7]. 

**Figure 16:**
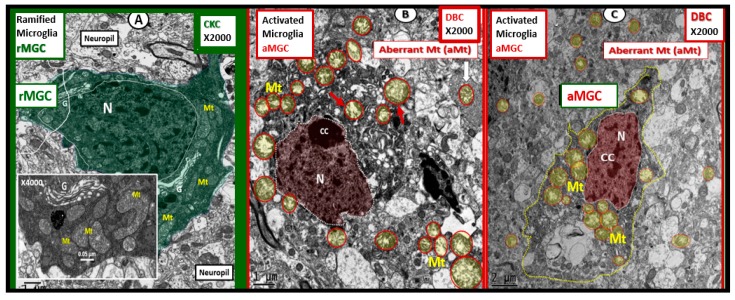
Activated microglia with aberrant mitochondria in diabetic *db/db* compared to ramified microglia in control non-diabetic models. Panel (**A**) illustrates the normal appearing ramified microglia (rMGC) in control CKC. Insert demonstrates the normal electron dense mitochondrial matrix with prominent crista. In contrast, panels (**B**) and (**C**) depict the activated amoeboid microglia cell (aMGC), a marked increase in aberrant mitochondria (Mt), and not the nucleus (N) chromatin condensation (CC) in the diabetic DBC models. G = Golgi; Mt = mitochondria; N = nucleus. Magnification ×2000; bar = 1 µm. Exploded insert magnification ×4000; bar = 0.05 µm. CC by 4.0 [6].

Recently, female diabetic *db/db* models were observed to primarily harbor aMGC in cortical layer III in the grey matter in contrast to rMGCs in controls. Additionally, these aMGCs had a marked increase in aberrant mitochondria (aMt) and their nuclei contained a definite increase in chromatin condensation (Figure 16) [6]. Importantly, empagliflozin (SGLT2 inhibitor) a glucose lowering treatment for 10 weeks ameliorated these abnormal remodeling changes in MGCs [6]. 

**Figure 17:**
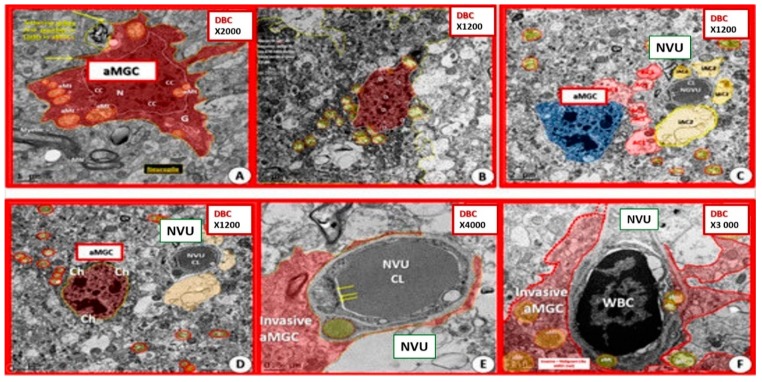
Activated microglia become invasive of the neurovascular unit in diabetic *db/db* models. Panels (**A**) and (**B**) portray activated microglia cells (aMGC) in diabetic DBC. Panels (**C**–**F**) depict the invasion of the neurovascular unit (NVU) by aMGCs in DBC. Note the frown-faced chromatin condensation in Panels C and D. Magnification ×2000 Panel A; ×1200 Panels (**B**–**D**); ×4000 panel E; ×3000 panel F with varying scale bars lower left. CC by 4.0 [6].

In addition, aMGC were demonstrated to be invasive of the NVU in the diabetic *db/db* models (Figure 17). 

While the study and knowledge of MGCs is expanding exponentially in regard to neuroinflammation and its relation to T2DM and LOAD, the author would like to direct those with beginning or ongoing interests in MGCs to read the following physiology review by Kettenmann H et al. [103]. Interestingly, the nine original postulates from the book chapter “Microglia” by Pio del Rio-Hortega in 1932 still hold true to this very time [104].

### 2.7. Oligodendrocyte and Myelin–Diabetic Gliopathy 

Oligodendrocytes (OLs), oligodendrocyte precursor cells (OPC) and oligodendrocyte lineage cells are specialized glial cells responsible for the synthesis, wrapping ensheathment, and compacting of myelin in myelinated axons [105,106,107]. OL-derived myelin additionally serves as a protective sheath in myelinated axons in order to provide for long-term axon integrity, maintenance and survival as well as increasing the speed of information transmission from neuron to neuron or regional bundles to distant bundles via white matter tracts as in optic nerves and corpus callosum. These white matter tracts are important for carrying/transferring large amounts of information from one region of the brain to distant regions and must rapidly transmit this information. This rapid transmission is primarily due to its compacted electron dense myelin sheaths. As a result, if there is any abnormal remodeling change there may be delays in the arrival of information to the more distant regional neurons with resulting cognitive impairment [108]. Of note, the newer technology of diffusion tensor imaging may lead the way into the future in regard to our better understanding of white matter tract abnormalities in T2DM and LOAD [108]. Certainly, it is already known that prediabetes and T2DM are associated with structural brain abnormalities including lacunar infarcts, white matter hyperintensities (WMHs), cerebral microbleeds (CMBs) and brain atrophy [109]. 

**Figure 18:**
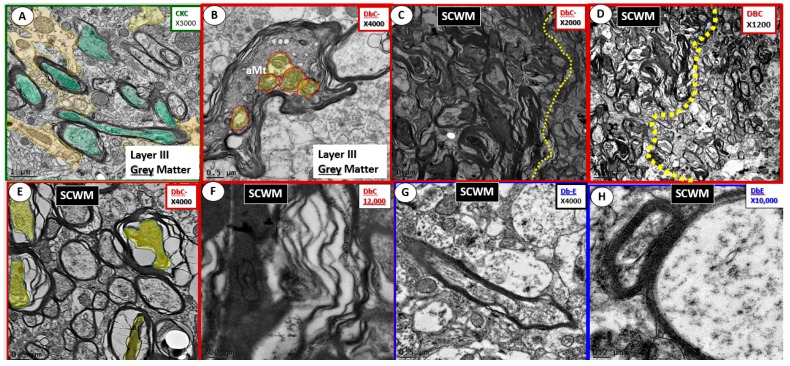
Myelin grey and subcortical white matter comparisons. Panel (**A**) illustrates normal electron dense myelin ensheathing axons in Layer III of the grey matter in control CKC models. Magnification ×3000; bar = 1 µm. Panels (**B**–**F**) depict the abnormal myelin remodeling in the diabetic *db/db* (DBC) models with panel B in grey and panels (**D**–**G**) in subcortical white matter transitional zone just beneath Layers I–VI in the subcortical white matter at various magnifications with scale bar of 0.5 µm (**B**); 1 µm (**C**); 2 µm (**D**); 0.05 µm (**E**); 200 nm (**F**); 0.5 µm (**G**); 0.2 µm (**H**). aMt = aberrant mitochondria; SCWM = subcortical white matter. CC by 4.0 [7].

Previous information demonstrating prominent remodeling changes of oligodendrocytes with increased nuclear chromatin condensation and volume and increased numbers of active myelination sites of the cytoplasm in subcortical transition zones beneath the cortical layers I–VI in the diabetic *db/db* models with known impaired cognition [7,82]. Marked dysmyelination has also been observed in outer myelin lamellae sheath with splitting, separation and ballooning with aberrant mitochondria in grey matter and similar myelin remodeling changes with marked myelin disarray and additional axonal collapse in transitional zones in DBC as compared to control CKC models [7], which were protected with the glucose lowering effects of empagliflozin (Figure 18) [7]. 

Additionally, Desai MK et al. have demonstrated that OL/myelin remodeling changes are an early manifestation of triple transgenic AD mice (3×Tg-AD) as they age and this could possibly also be pertinent to development of LOAD in humans [110]. This possibility may be even more relevant since MetS, obesity, insulin resistance and T2DM are associated with earlier OL/Myelin remodeling in *db/db* diabetic models [7]. Importantly, Ramos-Rodriguez JJ et al., demonstrated gross observable cortical brain atrophy and decreased brain weights in 26-week-old diabetic db/db mice [111]. Moreover, previous MRI studies in T2DM humans have been reported wherein, smaller total brain volumes and cortical grey matter and hippocampal atrophy was evident [112,113]. The ultrastructural images in this section and others throughout this review have been from a 20-week-old diabetic *db/db* models. Even though aging remains the strongest risk factor to develop LOAD and VAD, T2DM remains a risk factor for each of these dementias that indeed may be synergistic via microvascular (NVU) and white matter OL/myelin remodeling with impaired barrier function. 

As can be observed in the previous images each of the glia cells (AC, MGC and OL) may be associated with a diabetic gliopathy.

### 2.8. Neurovascular Unit and Barrier Functions

Earlier, in Section 2, it was mentioned that there may be three or four barriers provided by the capillary NVU depending if you count the EC BM and the PC inner and outer membrane separately or as continuous BM encasing both the EC and Pc and Pc foot processes (Pcfp). Recently, there has been a newer understanding of brain endothelial cells, NVU and BBB, in that, Kutuzov N et al. have examined the penetration of large (40 and 150 kDA dextran) and smaller molecular weight sized (376Da sodium fluorescein and 463Da Alexa flor) hydrophobic tracers from carotid artery into the brain to study passive transport from blood to brain [114]. They were able to elegantly demonstrate that the ecGCx is a significant first barrier by utilizing fast scanning two-photon microscopy via a cranial window and further, that the EC cytoplasm and its transport systems and adjacent tight and adherence junctions are the second barrier and that the endothelial cell, Pc and Pcfp BMs and ACs create yet a 3rd barrier due to differences in diffusion partition coefficients barrier properties on the brain side of the NVU. Furthermore, they suggested that these three sequences of diffusional constraints or barriers (ecGCx, endothelium and extravascular compartment) be termed the “Tripartite BBB” [114]. 

These functional diffusional barrier compartments coincide with the ultrastructural findings in this review with the exception that this review initially considered the endothelial BM as its own separate diffusion barrier because it was remodeled and thus four barriers vs. the tripartite barriers by Kutuzov N et al. Certainly any ultrastructural remodeling changes or alterations to the enGCx, EC and Pc BMs and ACs as a result of T2DM in previous images would have deleterious consequences to the integrity of the NVU due to a decrease in barrier functions with increased permeability and create an increased cellular vulnerability due to disruptions and loss of integrity to increase the risk and development of LOAD over time and aging.

## 3. Oxidative Stress: Reactive Oxygen/Nitrogen Species (ROS/RNS), Redox Stress and Aberrant Mitochondria (aMt) 

**Figure 19:**
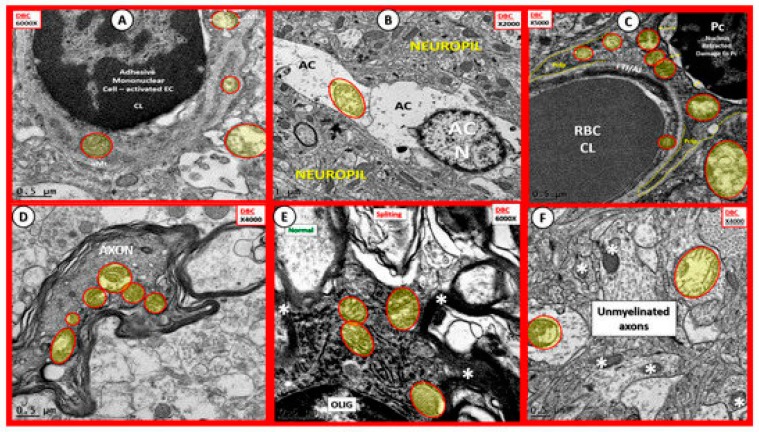
Aberrant mitochondria in endothelial cells, pericytes and foot processes, astrocytes, oligodendrocytes, myelinated and unmyelinated neurons in the diabetic DBC models. Panels (**A**–**F**) demonstrate that aberrant mitochondria (aMt) are found to be present in multiple cells in addition to activated microglia cells (aMGCs). The aMt are pseudo-colored in each of these panels (yellow outlined in red lines) in order to allow rapid recognition. Panels B and F are especially important since they demonstrate the aMt characterized by swollen mitochondria (Mt), loss of electron dense Mt matrix and crista. Panel A illustrates the aMt within the endothelial cells and surrounding aMGC. Panel B depicts aMt in ACs. Panel C demonstrates aMt in pericytes and foot processes (Pc and Pcfp). Panel D depicts aMt in a dysmyelinated neuronal axon. Panel E depicts aMt in an oligodendrocyte and Panel F illustrates aMt in an AC to the left and an unmyelinated axon on the right within the neuropil. Magnifications are noted in the upper part of each panel and scale bars are located at the lower left-hand side of all panels. Scale bars = 0.5 μm in all images except for panel B with scale bar = 1 μm. CC by 4.0 [5,6,7].

aMt were found to be markedly increased in aMGC in the diabetic db/db models in Section 2.6. and were also found to be present in other vascular mural cells (EC and Pc) of the NVU, AC, OL and neurons (myelinated and unmyelinated) in the grey matter cortical regions layer III and the transitional subcortical white matter regions of the diabetic *db/db* brain (Figure 19) [5,6,7,8].

**Scheme 3:**
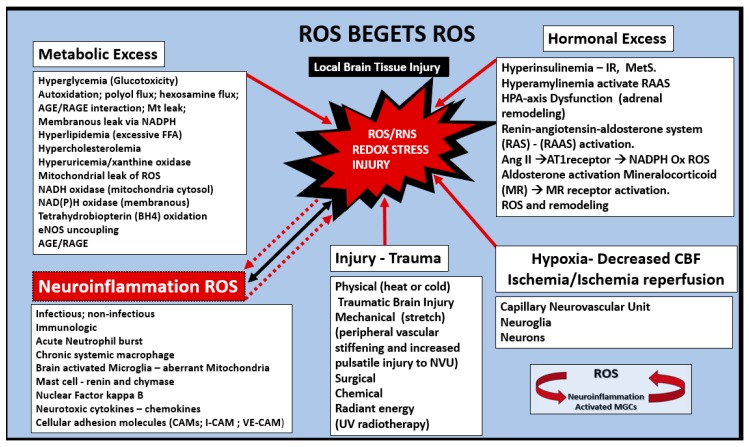
Reactive oxygen species (ROS) beget ROS. This figure illustrates how the metabolic excesses and hormonal excesses of the MetS interact to produce reactive oxygen/nitrogen species (ROS/RNS) and redox stress. Moreover, note how neuroinflammation, brain injury and hypoxia-ischemia and reperfusion may all work individually or synergistically to produce redox stress damage to the brain resulting in accelerated aging and neurodegeneration. At every downward arrow in the continuum of progression in Figure 5, ROS/NOS and redox stress are involved in the progression of age-related LOAD and support the oxidative stress hypothesis of LOAD. From obesity and insulin resistance of the MetS to T2DM to NVU remodeling to LOAD. AGE/RAGE = advanced glycation end products/ receptor for advanced glycation end products; Ang II = angiotensin II; AT1R = angiotensin type 1 receptor; eNOS = endothelial nitric oxide synthase; HPA = hypothalamic pituitary adrenal; MC = mast cell; MGCs = microglia cells; NADPH – NADPH Ox = reduced nicotinamide adenine dinucleotide phosphate oxidase; NVU = neurovascular unit; NF-kB = nuclear factor- kappa B; RAS = renin angiotensin system; RAAS = renin angiotensin aldosterone system; ROS/RNS = reactive oxygen species/reactive nitrogen species.

Oxidative nitrosative Stress (ROS/RNS) seems to be of extreme importance at every turn of events or downward arrow from obesity to T2DM to LOAD in the continuum of progression Section 1.6 (Figure 5). ROS beget ROS independently and via previously presented aMGC Section 2.6 (Figure 16 and Figure 17). The neuroinflammatory aMGCs enzymatic iNOS, NADPH Ox and Mt-derived excessive ROS due to aberrant mitochondria may interact and fuel other sources of ROS/NOS such as: Metabolic excess, hormonal excess, renin angiotensin aldosterone system activation, inflammation systemic or neuroinflammatory, hypoxia-ischemia, ischemia-reperfusion, eNOS uncoupling associated with EC dysfunction as in the MetS in Section 1.2 (Figure 2). These accumulating ROS/NOS of oxidative stress may be synergistic in promoting brain injury with subsequent response to injury remodeling changes in structure and functional abnormalities as in Scheme 3. 

### Oxidative Nitrosative Stress (ROS/NOS): Aberrant Mitochondrial-Derived ROS/NOS Leakage in T2DM End-Organ Complications 

**Scheme 4:**
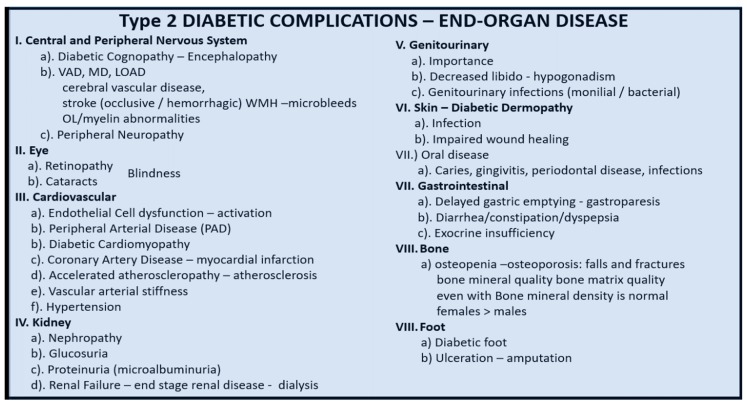
Type 2 diabetes mellitus and end-organ remodeling complications. Aberrant mitochondria (aMt) are a central figure in most, if not all, of the end-organ complications. Diabetic end-organs may all have problems with decreased energy production (ATP) and excessive Mt-derived oxidative stress (ROS) production due to abnormalities in structure as well as function. LOAD = late-onset Alzheimer’s disease; MD = mixed dementia; OL = oligodendrocyte; VAD= vascular dementia; WMH = white matter hyperintensities.

Previous studies have demonstrated an important role of mitochondria in diabetic end-organ complications (Scheme 4). 

In general, mitochondria lie at the intersection of many critical cellular pathways involving energy substrate metabolism (ATP generation), calcium homeostasis (acting as a calcium sink), reactive oxygen species (ROS) generation and apoptosis signaling. Furthermore, in healthy cells mitochondria exist as a dynamic reticulum network (in constant fusion and fission) that move around within the cytoplasm assisted primarily by a microtubule actin motor network [115]. Mitochondria are primarily formed within the soma and migrate distally down the axon to high energy requiring synapses and if they undergo aberrant mitochondrial (aMt) remodeling changes, they migrate antegrade for repair via (fission and fusion mechanisms) or undergo mitophagy. Thus, if there is impaired autophagy or mitophagy or impairment in microtubule transport due to paired helical fragments and or neurofibrillary tangles even in the early stage of tau formation there may be impaired mitochondrial movement and repair, i.e., hyperphosphorylated and aggregated tau appears to damage the axonal transport, leading to abnormal mitochondrial structural defects in synapses [116]. 

In many of the end-organs that have been previously studied by the author with obesity-genetic or diet induced, insulin resistance, MetS, impaired glucose tolerance or overt T2DM, the mitochondria have demonstrated either functional and/or an ultrastructural abnormalities, suggesting that oxidative stress may be at the very core of diabetic end-organ complication damage similar to those found in the brain [117,118,119,120,121,122,123,124,125,126,127,128,129]. Thus, ultrastructural aMt in peripheral or central CNS tissues may allow one to strongly suspect decreased ATP generation–hypometabolism, excessive aMt-derived ROS production leakiness and oxidative stress. Of interest is that patients and genetic models with LOAD and AD, respectively, experiece mitochondrial dysfunctions (aMt) that are associated with bioenergetic deficits and oxidative stress that occur early and may promote Aβ and Tau pathologies [130]. 

**Scheme 5:**
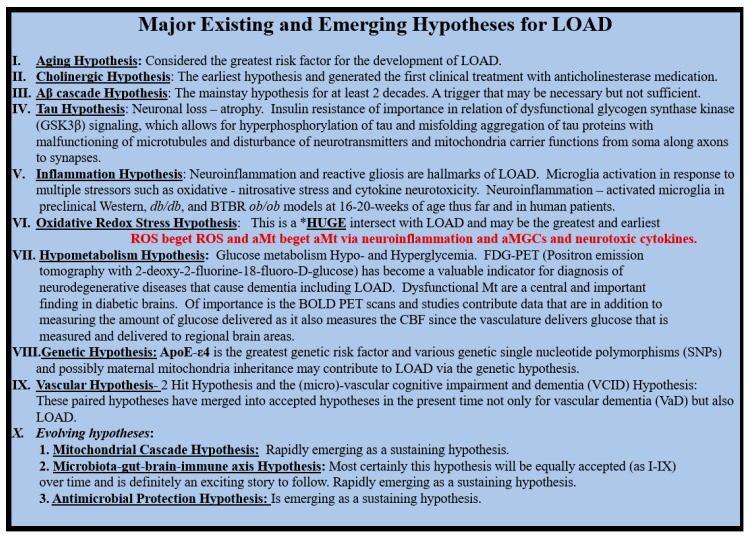
Major existing and emerging hypotheses for late-onset Alzheimer’s disease (LOAD). AD = Alzheimer’s disease; APOE-ε4 = apolipoprotein E epsilon 4; BOLD = blood-oxygen-level-dependent imaging; CBF = cerebral blood flow; LOAD = late-onset Alzheimer’s disease; MGC = microglia cell; PET = positron emission tomography; ROS = reactive oxygen species; T1DM = type 1 diabetes mellitus; T2DM = type 2 diabetes mellitus; VaD = vascular dementia.

Nunomura A et al. have demonstrated that oxidative damage is one of the earliest events and furthermore as Aβ deposition increases and LOAD progresses there is an associated decrease in oxidative damage injury. They utilized measurements in both neuronal oxidized RNA 8-hydroxyguanosine (8OHG) and the oxidized amino acid nitrotyrosine (3-NT) in LOAD patients. They also demonstrated via the 8OHG immunogold studies, which labeled the cytoplasmic ribosomal endoplasmic reticulum and not the nucleus regions of the neurons. The authors suggested these findings may be due to an increased antioxidant response to Aβ and tau formation [131]. Importantly, if there were chronic ongoing oxidative stress associated with concurrent and/or preexisting ongoing chronic MetS, insulin resistance and T2DM then antioxidant depletion would be more plausible. Swerdlow RH et al. during a decade (2004–2014) progressed the concept of the mitochondrial hypothesis such that it has now been included in the evolving hypotheses of LOAD (Scheme 5) [132,133,134,135].

## 4. Major Existing and Emerging Hypotheses for LOAD 

LOAD is an age-dependent, heterogeneous, multifactorial and neurodegenerative disorder functionally characterized early-on at its onset by mild cognitive impairment with a progressive cognitive decline to become the sixth leading cause of death. Pathologically, LOAD may be characterized by progressive deposition of extracellular amyloid-β (1–42) (Aβ) neuritic plaques derived from amyloid precursor protein (APP), vascular deposition of amyloid-β (1–40) in the arteriole vascular media and paired helical fragments (PHF) and neurofibrillary tangles (NFT) derived from abnormal hyperphosphorylation of tau proteins primarily within the neuron. 

Aβ is formed after sequential cleavage of APP by the proteolytic enzymes β- and γ-secretases. The γ-secretase cleavage at the C-terminal end of the transmembrane region of APP generates a number of isoforms of 36–43 amino acid residues. The most abundant isoforms are Aβ (1-40), which are found primarily in cerebral amyloid angiopathy and Aβ (1–42) in neuritic plaques. Moreover, varying lengths of oligomers of Aβ have been found to be the toxic form of Aβ rather than the Aβ neuritic plaques [136]. 

Tau is a neuron microtubule associated protein that is essential for proper function. The microtubules are associated with the cytoskeletal actin motor unit elements of neurons and are essential for the mobility of neurotransmitter vesicles and mitochondria cellular movement along with other organelles within neurons during their long passage from the soma to the dendritic synapses to provide synaptic transmission from one neuron to another. In LOAD tau becomes hyperphosphorylated and is known to disrupt normal microtubule functions with impaired transport of neuronal cellular organelles to dendritic synapses and, if necessary, to transport damaged mitochondria antegrade to the soma regions to undergo macro-autophagy or mitophagy. Tau is thought to become hyperphosphorylated and in this hyperphosphorylated state it becomes polymerized into paired helical filaments (PHF) admixed with straight filaments, which ultimately form neurofibrillary tangles (NFTs) and the second hallmark lesion of misfolded proteins along with Aβ that is associated with LOAD. [137]. Currently it is thought that Aβ formation and deposition occurs earlier than the PHF and NFT of tau. Additionally, PHF and NFT of misfolded tau correlate more closely with the clinical progression of the disease in LOAD [136,137]. 

Over the last century, since Alois Alzheimer made his initial discovery in a younger early onset 50-year-old female patient suffering from dementia in 1906, there have emerged numerous hypotheses regarding the development of LOAD [138]. These numerous major and emerging hypotheses (Scheme 5) have developed from many different laboratories and multiple researchers and will undoubtedly continue to grow and/or change as new information comes forward, but for now these are currently felt to be the best candidate hypotheses. 

### 4.1. Endothelial Cell Activation–Dysfunction and Impaired Cerebral Blood Flow (CBF) in Diabetic db/db Models: Hypometabolism (VII) and Vascular (IX) Hypotheses Elaborated 

The brain receives 20% of the cardiac output and 20% of the body’s oxygen and glucose in order to maintain proper homeostasis. CBF is responsible for the delivery of essential oxygen and glucose and therefore healthy blood vessels are essential and, if interrupted, damage may occur to cerebral neurons in minutes. This estimated 400-mile trek of capillary NVUs allows the cellular elements to be perfused and when there is increased neuronal activity this neurovascular coupling allows for increased CBF to provide the necessary nutritional supply of glucose and oxygen, which is dependent on intact NVU coupling and functional hyperemia [139]. Thus, if there is any dysfunction or interruption of CBF to supply the increased neuronal activity the neurons suffer initially with dysfunction and later neurodegeneration due to oligemia–ischemia. 

EC function plays a huge role in maintaining the competence of NVU coupling and CBF in the brain via functional hyperemia. EC dysfunction or activation is associated with impaired NO production via eNOS enzyme uncoupling due to oxidative stress, decreased tetrahydrobiopterin (BH4), increased vasoconstrictors such as endothelin-1 (ET-1), angiotensin II (Ang II) and disturbed regulation of inflammation and thrombosis. Since NO is known to be an inhibitor of platelet aggregation and leukocyte adhesion, EC activation has detrimental effects on cerebrovascular–NVU functions and CBF [140]. EC dysfunction–activation (Figure 2) was presented in Section 2 and is known to be present as an early abnormality in MetS and T2DM. EC activation in the NVU due to attenuation and or loss of endothelial glycocalyx, as well as, impaired nitric oxide generation and eNOS enzyme uncoupling with decreased NO bioavailability could contribute to NVU uncoupling and decreased CBF [140]. 

**Figure 20:**
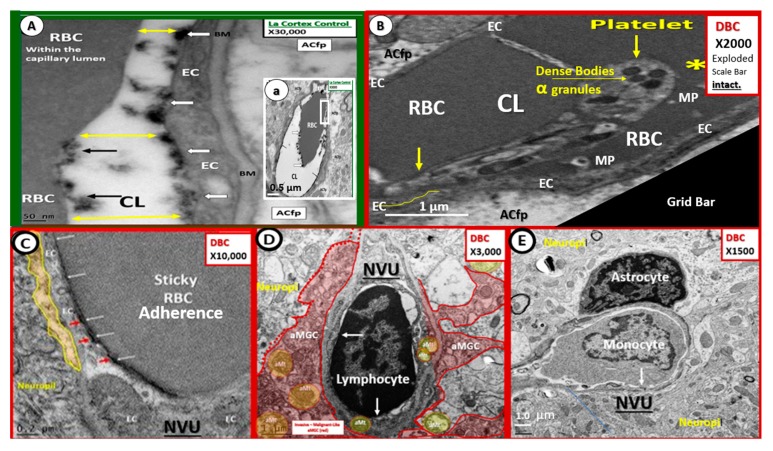
Adherent red blood cells, leukocytes and activated platelets to activated endothelial cells in *db/db* models. Panel (**A**) depicts an intact endothelial glycocalyx (ecGCx) on both the intraluminal red blood cell (RBC) and the luminal surface of the endothelial cell (EC) with lanthanum nitrate staining (arrows) with magnification ×30,000; scale bar = 50 nm in a non-diabetic control C57B6 mouse model. Insert (a) demonstrates this same capillary neurovascular unit (NVU) with intraluminal RBC with lower magnification ×3000; scale bar = 0.5 µm to depict the entire capillary NVU. Note that the EC and RBC are each decorated by an extremely electron dense protective surface coating of an ecGCx (arrows) that are separated and not adherent in control models with pre-sacrifice lanthanum nitrate infusion that has been found to be lost or shed in diabetic models (such as in the *db/db* and *ob/ob* models—not shown). Panel (**B**) depicts an adherent activated platelet (arrows and yellow line lower left) to the EC and note the elongation of this platelet suggesting activation in addition to its adhesion to the capillary NVU endothelial cell. Magnification ×2000 enlarged in paint program with intact scale bar = 1 µm. Panel (**C**) illustrates adhesion of a RBC to the EC in the *db/db* diabetic DBC model with an electron dense adhesion plaque (electron dense (arrows) of the NVU. Magnification ×10,000; scale bar = 0.2 µm. Panels (**D**) and (**E**) depict the tight adherence of leukocytes to the endothelium (adherence points depicted with arrows) in the diabetic *db/db* DBC models. A lymphocyte in panel D and a monocyte in panel E. This tight adherence of RBC and leukocytes and activated platelet are representative of an activated endothelium in the *db/db* diabetic DBC models. Various magnifications are utilized to demonstrate the adherence. Magnification ×3000; bar = 1 µm (panel D); magnification ×1500; bar = 1.0 µm (panel E). Panels C and D CC by 4.0 [5].

Altered platelet, red and white blood cell rheology is known to be present in T2DM [141]. Adherence of activated platelets, erythrocyte and leukocytes are known to be associated with EC activation in preclinical models and humans with T2DM [142,143]. Previous studies in the diabetic female T2DM *db/db* models have demonstrated major remodeling changes to the EC as a result of EC activation in the NVU, which consisted of platelets, red blood cells (RBCs), leukocyte adherence in addition to the well-known fibrin network dysfunctional abnormalities (Figure 20). 

The adhesion of RBCs, leukocytes and activated platelets to the NVU activated endothelium observed in the obese insulin resistant *db/db* diabetic DBC models as observed in Figure 20 would definitely result in increased inflammation and oxidative stress but equally important would be their physical obstruction to CBF and decreased distal perfusion and resulting ischemia to neurons due to a stalling of CBF. 

Just as there were adherent and stalled neutrophils in the study by Cruz Hernández JC et al. the *db/db* DBC diabetic mice have demonstrated adherent stalled RBCs, leukocytes and platelets. So, in addition to the impaired CBF that may be associated with AC retraction and loss of neurovascular coupling and impaired vascular functional hyperemia there may also be adherent stalled platelets, RBCs and leukocytes due to adhesion to an activated and dysfunctional endothelium. As a result of our findings regarding the AC detachment and adhesion of platelets, RBCs and leukocytes in the diabetic *db/db* models we can now demonstrate at least two morphological ultrastructural changes in the *db/db* diabetic model that could interfere with CBF that appear to be protected with empagliflozin [8]. This type of oligemia and decreased CBF in the *db/db* diabetic mouse model would certainly accelerate the remodeling changes and contribute to the increased risk that would be associated with pre-existing T2DM on the age-related development of LOAD. Additionally, impaired CBF as measured by cerebral glucose metabolic rate (CMRglu) by fludeoxyglucose F 18-positron emission tomography (FDG-PET) are known to be associated with LOAD risk and even observed prior to the onset of dementia [144,145,146,147]. Thus, brain hypoperfusion and impaired CBF may participate in the pathogenesis/pathophysiology of neurodegenerative diseases such as in LOAD with underlying T2DM. 

A brief synopsis of some of our findings certainly tie into the preceding paragraphs in that, the five major conditions/categories as listed in the abstract consisted of i. aging; ii. metabolic (hyperglycemia and advanced glycation end product and receptor AGE/RAGE interaction resulting in oxidative stress and inflammation in addition to insulin resistance as a linchpin between T2DM and LOAD); iii. oxidative stress; iv. inflammation; v. vascular (extra and intracranial macrovascular, vascular stiffening and NVU microvascular disease) all come into play when discussing the increased risk of LOAD with underlying T2DM and the phenomenon of impaired CBF. Additionally, in the *db/db* (DBC) diabetic model we have learned that there is microvascular disease of the NVU with EC activation with platelet, RBC and WBC adherence resulting in decreased CBF due to cellular adhesion resulting in stalling of CBF. Additionally, attenuation and/or loss of the BBB TJ/AJ, thickening of the BM, detachment of the AC with impairment of functional hyperemia and neurovascular uncoupling between active neurons and dilation of the NVU in T2DM could markedly increase the vulnerability of these cells as age-related LOAD begins to develop and could contribute to the brain atrophy noted by Ando Y [63].

## 5. Neuropathological Changes in LOAD

The neuropathologic substrate of LOAD consists of extracellular neuritic plaques (Aβ 1–42) and CAA (Aβ1–40) and intracellular–intraneuronal hyperphosphorylated misfolded microtubule-associated tau proteins [148]. Despite the newer sophisticated diagnostic procedures and tests, autopsy and neuropathological examination continue to remain the ‘gold standard’ for the definitive diagnosis of LOAD. The only visible gross finding in LOAD is cortical atrophy and enlargement of the lateral ventricles; however, this is not specific for LOAD and could include many other neurodegenerative diseases, but this finding certainly raises a red flag to the neuropathologist. Numerous regions within the brain are affected by the neuritic plaques and neurofibrillary tau tangles; however, the entorhinal cortex, the CA1 and subicular regions of the hippocampal regions are in particular heavily involved along with other regions such as the amygdala, and the deeper layers (layers III, V and superficial VI) of the neocortical regions of the brain. The extent and distribution of neurofibrillary tangles in cases of LOAD correlate with both the degree of dementia and the duration of illness as well as staging [149] and have been thought of as one of the cardinal histopathological lesions along with neuritic plaques of LOAD. There has also been demonstrated to be a synaptic loss of neurons in LOAD (up to 45%) and this finding is important to the neuron to neuron communication and synaptic loss is felt to contribute to the cognitive impairment found in these individuals along with cerebral atrophy. Even though LOAD is the sixth leading cause of death, it is often not the direct cause of death and many of these patients may die from other associated diseases such as sepsis, pneumonia, traumatic falls with injury and cardiovascular diseases including stroke due to the older age of these patients with multiple comorbidities and frailties. The most commonly associated condition with LOAD is stroke and ischemic infarction. Additionally, another co-morbid condition, Parkinson’s disease with or without Lewy bodies, is often associated with clinical diagnosis of LOAD at the time of neuropathologic study. While the above may be somewhat of a superficial sketch of the neuropathological findings, it does add to the overall knowledge and background of this important topic [148]. Additionally, these previous observations speak out strongly for the importance of mixed dementias as discussed in Section 1.5 (Figure 4).

## 6. Discussion and Concluding Remarks 

MetS and T2DM are known to be associated with macro and microvascular end-organ complications including accelerated atherosclerosis, peripheral neuropathy, retinopathy, nephropathy and cardiomyopathy for decades. In contrast, the brain has been studied as an end-organ of T2DM for a briefer time period. While the mechanisms implicated have not been completely elucidated it is now known that cognitive impairment, VaD, stroke, anxiety/depression and LOAD are related to T2DM. Additionally, connecting astrocytes and their unique ultrastructure and functions are signaled by the activity of regional neurons providing neurovascular coupling and functional hyperemia to increase cerebral blood flow in times of need. Thus, the NVUs with their readily identifiable ultrastructure has directed this review to heavily focus on both its structure and its function in order to autoregulate its vascular supply. 

Prior to this time the BBB along with its necessary TJ/AJ held the main role of protecting the neurons but now we understand that there is more to this highly dynamic structure as we study the ultrastructural remodeling of the NVU that facilitates crosstalk between its cells. Once this important NVU structure is injured and remodeled by T2DM, the brain may be deficient in its proper response to injury function and allow synaptic and neuronal dysfunction, with neurodegeneration and atrophy. 

The risk for stroke or dementia is great for both sexes, i.e., at age 65, one out of four men and one out of three women will have an increased risk of developing stroke, dementia or both [150]. Additionally, when the first baby boom generation began turning 65 years of age at a rate of approximately 10,000 per day in 2011, they were estimated to be just under 77 million strong in the United States population (https://www.census.gov/prod/2014pubs/p25-1141.pdf accessed 10 July 2019). 

Since T2DM and LOAD are both age-related and chronic diseases, it is not unusual for them to co-occur along with cerebro-cardiovascular disease in global aging societies. Both have multifactorial causations and risks with insulin resistance as a linchpin between the two disparate diseases. A great deal remains unknown; however, a wonderful discussion regarding what is and what is not known regarding brain insulin resistance (BIR) can be found and is strongly suggested [151,152,153]. 

Peripheral insulin resistance is a definite core feature of T2DM that is rapidly emerging as a core feature in LOAD as BIR, which may be defined as the failure of brain cells to respond to insulin [153,154]. A better understanding of BIR is forthcoming, since intranasal insulin is to be studied in human clinical trials [155]. Intranasal insulin has been able to improve multiple systems that are known to be involved with LOAD like impaired glucose uptake, memory, focus and activities of daily living in multiple studies without causing hypoinsulinemia [156]. Six intranasal insulin treatment trials have been rigorously reviewed in regard to MCI and LOAD by Avgerinos KI et al. [157,158,159,160,161,162,163]. Additionally, the combination of T2DM and LOAD suggest that the two disparate diseases may be synergistic when they co-occur and demonstrated the greatest decrease in insulin signaling proteins [163]. Recently Liu Y et al. found that the brain PI3K-AKT signaling pathway from autopsied frontal cortices demonstrated the greatest PI3K–AKT signaling impairment in individuals with both T2DM and LOAD [163]. Further, they found in T2DM and LOAD patients there was a decrease in insulin receptors, IRS-1 total, PI3K(85) total–phosphorylated (p)PI3K(p85), PDK1total–(p)PDK1, AKT total–(p)AKT, GSK-3βtotal–(p)GSK-3β (allowing hyperphosphorylation of tau and PHF and NFT) and increased Calpain-1, which could allow for increased degradation of these decreased insulin signaling proteins [163]. Additionally, they found that there were decreases in glucose transporters, namely GLUT1 (endothelial GLUT) and GLUT3 (neuronal GLUT) as well as decreased HIF-1 and O-GlcNAcylation, which correlated with increased hyperphosphorylation of tau in short time post-mortem AD brains (<3 h). These findings strongly support the notion that GLUT1 and GLUT3 deficiencies could cause impaired brain glucose uptake and/or metabolism, which could contribute to neurodegeneration in correlation to a decrease in O-glcNAcylation and hyperphosphorylation of tau in AD [164]. 

The following two additional emerging hypotheses may be ‘game changers’ regarding the novel treatments of LOAD by repurposing insulin and insulin sensitizing medication and aiding in the understanding of the development and/or the continuum of progression of T2DM to LOAD.

### 6.1. Metabolic Hypothesis: Hyperglycemia and Hyperinsulinemia 

Hyperglycemia-glucotoxicity and hyperinsulinemia of T2DM and MetS with peripheral insulin resistance (PIR) and BIR may be extremely injurious to brain metabolic functioning and brain end-organ remodeling associated with the increased risk in the development of LOAD [165]. 

This evolving hypothesis allows one to consider the possible impact of excessive amounts of amyloidogenic amylin (islet amyloid polypeptide—IAPP)–hyperamylinemia to be deposited in the brain’s perivascular and extracellular matrix. Amylin is co-synthesized, co-packaged and co-secreted from the islet pancreatic beta cell’s insulin secretory granule due to peripheral insulin resistance (PIR) [166,167]. Amyloidogenic amylin may be deposited as amyloid amylin (Aα) and eventually function as a possible niche region in the brain for cross-seeding with Aβ and allow Aβ and Aα to undergo mixed perivascular and extracellular co-deposition in addition to Aα independent deposition in the perivascular regions and extracellular matrix, which may possibly interfere with Aβ efflux or clearance [168,169,170,171,172,173]. The Aα hypothesis might also lead to the use of an amylin analog (pramlintide) for clinical study that has already been approved and utilized to treat type 1 and T2DM and known to improve glucose levels [174]. Incidentally, pramlintide has been reported to improve the feeling of “well-being” by those having used it in the original treatment trials (authors personal communication in 2004) [175]. 

Moreover, fibrinogen is one of the biomarkers of inflammation and an important risk factor for many cardiovascular and cerebrovascular diseases including T2DM and LOAD [176]. Misfolded proteins and their deposition are important to the development of LOAD. Amyloid fibrinogen-fibrin (AFib) due to activated platelets and their membrane amyloid precursor protein within the NVU may allow for the fifth misfolded protein in LOAD, plus elevated fibrinogen is known to be associated with Met and T2DM [176]. Currently, there are at least five known and distinct misfolded proteins consisting of Aβ (1–40), Aβ (1–42), tau (NFT), amyloid amylin (Aα) and now AFib that contribute to Aβ (1–42) via the activated platelets and systemic fibrinogen. 

### 6.2. The Antimicrobial Protection Hypothesis: Antimicrobial Peptide(s) (AMP) 

Aβ is known to be an antimicrobial peptide [177]. Previous infections such as *Human alphaherpesvirus 1* (HSV-1—viral), *Chlamydia pneumoniae* (bacterial), *Borrelia burgdorferi* or *recurrentis* (*spirochete*) plus other pathogens or the ongoing chronic sterile inflammatory process (activated MGCs) have all been implicated in activating AMPs [176,177]. Interestingly, this infectious hypothesis or antimicrobial protection hypothesis is somewhat reminiscent of the *Helicobacter pylori* (gastritis and duodenal ulcer story) and certainly an emerging hypothesis to follow closely, while keeping in mind the potential of antiviral and antibacterial drug repurposing [178,179]. One such repurposing drug is minocycline, because it is known to pass the BBB and is also known to be anti-inflammatory and a matrix metalloproteinase inhibitor [180]. 

The ongoing chronic sterile inflammatory process (activated MGCs) as occurs in LOAD and T2DM may result in excessive AMP-APP production of Aβ (1–42) due to activation of excessive APPs. The chronic sterile inflammatory process may be perceived as a pathogenic-like threat, which results in increased APP synthesis and cleavage to oligomeric forms of Aβ (1–42) to form amyloid fibrils in order to entrap pathogens and serve as an innate immune pathway as a response to a perceived active infection or a sterile inflammatory response. This emerging antimicrobial protection hypothesis does not interfere with the existing Aβ cascade hypothesis; however, it does add a novel dimension to this exiting hypothesis and may suggest that an innate immune-mediated inflammation could propagate LOAD, Aβ(1–42) deposition and ongoing neurodegeneration [181,182,183]. Additionally, this could allow for the consideration of antibacterial, anti-spirochetal and antiviral treatments if an underlying source of infective agent could be identified via past medical history of previous infections or current diagnostic findings. 

The NVU concept was formalized at the 2001 Stroke Progress Review Group meeting of the National Institute of Neurological Disorders and Stroke and the NVU has definitely come of age [184]. The NVU concept allows for the dynamic interplay or coupling between neural activity and cerebral blood flow and functional hyperemia that forms the basis of functional magnetic resonance imaging, which measures blood oxygenation level dependent (BOLD) images to evaluate human individuals clinically and preclinical rodent models. NVU function may now be considered a multidimensional process involving signals from multiple cells that engage in distinct highly orchestrated signaling pathways and effector systems across the cerebrovascular capillary mesh-like network of NVUs in a symphonic-like process. Additionally, the NVU with its BBB–blood–brain interface performs as both an endocrine target and an endocrine secretory tissue and therefore, should also be viewed as an endocrine-like organ between the CNS and peripheral tissues, since it is capable of autocrine, paracrine and endocrine-like functions [185]. Furthermore, the NVUs tripartite BBB functions along with its interface functions, which incorporates signaling to its own supporting cellular network via crosstalk with Pcs, ACs, MGCs, OLs and neurons provide a vital link to CNS homeostasis. 

**Figure 21:**
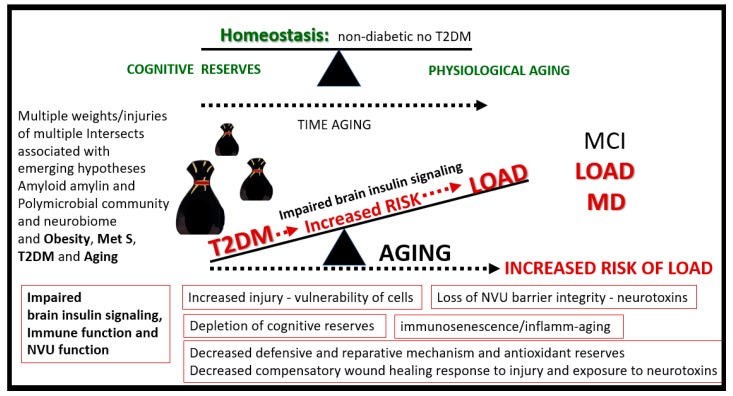
T2DM tips the balance for the development of LOAD. The multiple weights of injuries, metabolic insults, impaired brain insulin signaling, immune function, NVU dysfunction, intersects, ongoing chronic wounding of obesity, MetS, T2DM, aging and the increased burdensome weight of wounding injuries (multifactorial insults and injuries) set in motion the multiple wound healing responses to injury mechanisms with resulting increases in cellular vulnerability along with impaired signaling mechanisms for the development and increased risk of age-related LOAD. LOAD = late-onset Alzheimer’s disease; MCI = mild cognitive impairment; MD = mixed dementia; MetS = metabolic syndrome; NVU = neurovascular unit; T2DM = type 2 diabetes mellitus.

The multiple weights of multiple cellular injuries resulting in marked NVU ultrastructural remodeling in T2DM along with the emerging hypotheses of the metabolic hypothesis and antimicrobial protection hypothesis may increase the cellular vulnerability and tip the balance for the brain to develop an increased risk of LOAD (Figure 21). 

Throughout this review multiple efforts have been made to comprehensively utilize supportive ultrastructural TEM images from the cortical grey matter capillary NVUs of layer III and its constitutive cells from the obese, insulin resistant, diabetic *db/db* preclinical mouse model.

For most of adult life brain neurons do not experience exposure to peripheral neurotoxins; however, the aged brain and especially those individuals who develop T2DM in mid-life and older age groups experience exposure to peripheral neurotoxins as a result of the NVUs loss of barrier integrity. Brain neurons are not properly equipped to respond to increased exposure to peripherally derived neurotoxins and therefore remain dependent on an intact NVU. Therefore, the ultrastructural NVU and glia cellular remodeling changes as shared in this *db/db* diabetic model along with the associated functional changes demonstrate the important role of how structure and function are tightly related to the importance of obesity, aging, shared risks and the continuum of progression in regards to the presence of T2DM and the increased risk of LOAD. 

In conclusion, as T2DM and LOAD merge in our aging society they may form a “bottleneck” of senior citizens with an increased risk of co-occurrence dementias over the next two decades due to global aging. This conundrum will undoubtedly create a strain on our healthcare system, a financial burden to our societies and much stress to individual caregivers and families. The brain parenchyma and its vascular supply are the structural and functional substrate of the mind, which allow each of us to have a unique brain print. 

## Abbreviations

Aα	amyloid alpha
AC	astrocyte
Aβ	amyloid beta
ACfp	astrocyte foot processes
AD	Alzheimer’s disease
AFib	amyloid fibrinogen/fibrin
AGE	advanced glycation end products
AMP	antimicrobial peptide(s)
aMt	aberrant mitochondria
APP	amyloid precursor protein
aMGC	activated microglia cell
BB	baby boom generation following WWII
Boomer	baby boom generation following WWII
BBB	blood–brain barrier
BIR	brain insulin resistance
BM	basement membrane
CAA	cerebral amyloid angiopathy
CBF	cerebral blood flow
CKC	C57B6-J control models
CSF	cerebral spinal fluid
CVD	cerebro-cardiovascular disease
DBC	diabetic *db/db* models
DC	diabetic cognopathy
DIO	diet induced obesity
EC	endothelial cell
ecGCx	endothelial cell glycocalyx
eNOS	endothelial nitric oxide synthase
FFA	free fatty acids
HPA	hypothalamic–pituitary–adrenal (HPA) axis
H and E	hyper and excess phenomenon
HSV-1	herpes simplex virus type 1
IAPP	islet amyloid polypeptide
IR	insulin resistance
LOAD	late-onset Alzheimer’s disease
MC	metabolic cognopathy
MD	mixed dementia
MetS	metabolic syndrome
MGC	microglia cell
MT	microtubule
NO	nitric oxide
NFT	neurofibrillary tangles
NVU	neurovascular unit
OL	oligodendrocyte
Pc	pericyte
Pcfp	pericyte foot process
PHF	paired helical fragments
PIR	peripheral insulin resistance
RAAS	renin–angiotensin–aldosterone system
RAGE	receptor for advanced glycation end products
RBC	red blood cell
rMGC	ramified microglia cell
ROS/RNS	reactive oxygen species/reactive nitrogen species
T2DM	type 2 diabetes mellitus
Tau	microtubule associated protein
TJ/AJ	tight junctions/adherens junctions
VaD / VAD	vascular dementia
VCID	vascular contributions of impaired cognition and dementia
VSMC	vascular smooth muscle cell
WBC	white blood cell
